# Loss of ERα induces amoeboid-like migration of breast cancer cells by downregulating vinculin

**DOI:** 10.1038/ncomms14483

**Published:** 2017-03-07

**Authors:** Yuan Gao, Zhaowei Wang, Qiang Hao, Weina Li, Yujin Xu, Juliang Zhang, Wangqian Zhang, Shuning Wang, Shuo Liu, Meng Li, Xiaochang Xue, Wei Zhang, Cun Zhang, Yingqi Zhang

**Affiliations:** 1 State Key Laboratory of Cancer Biology, Biotechnology Center, School of Pharmacy, The Fourth Military Medical University, 169 Changle West Road, Xi'an 710032, China; 2 Department of Vascular and Endocrine Surgery, Xijing Hospital, The Fourth Military Medical University, 127 Changle West Road, Xi'an 710032, China

## Abstract

Oestrogen receptor alpha (ERα) is a well-known target of endocrine therapy for ERα-positive breast cancer. ERα-negative cells, which are enriched during endocrine therapy, are associated with metastatic relapse. Here we determine that loss of ERα in the invasive front and in lymph node metastasis in human breast cancer is significantly correlated with lymphatic metastasis. Using *in vivo* and *in vitro* experiments, we demonstrate that ERα inhibits breast cancer metastasis. Furthermore, we find that ERα is a novel regulator of vinculin expression in breast cancer. Notably, ERα suppresses the amoeboid-like movement of breast cancer cells by upregulating vinculin in 3D matrix, which in turn promotes cell–cell and cell–matrix adhesion and inhibits the formation of amoeboid-like protrusions. A positive association between ERα and vinculin expression is found in human breast cancer tissues. The results show that ERα inhibits breast cancer metastasis and suggest that ERα suppresses cell amoeboid-like movement by upregulating vinculin.

## Abstract (web summary)

Estrogen receptor alpha (ERα)-negative cells, which are enriched during endocrine therapy, are associated with metastatic relapse of breast cancer. Here the authors show that ERα inhibits breast cancer metastasis and suggest that ERα suppresses the amoeboid-like migration of breast cancer cells by upregulating vinculin.

The pathogenesis of breast cancer is associated with oestrogen receptor alpha (ERα), which is activated by sex hormones and contributes to the aberrant proliferation of breast cancer cells^1^,^2^. The classical mechanism of ERα action involves regulating the transcription of oestrogen-responsive genes by binding to the oestrogen-responsive element (ERE) within the promoters of the target genes^3^,^4^.

Endocrine therapy with selective oestrogen receptor modulators, such as tamoxifen^5^, has been widely used to antagonize ERα in breast cancer tissues^6^. However, tamoxifen appears to decrease the risk of ERα-positive contralateral breast tumours and to increase the risk of ERα-negative contralateral tumours^7^. Therefore, lost expression of ERα during adjuvant endocrine treatment for ERα-positive breast cancer allows for resistance to common adjuvant endocrine therapies and is associated with ERα-negative metastatic relapse^7^. Nevertheless, how ERα loss is associated with metastasis remains to be elucidated, particularly in a three-dimensional (3D) environment, which can better mimic human breast cancer metastasis *in vivo*.

The migration of invasive cells *in vivo* consists of the mesenchymal mode, in which invasive cells are elongated and require pericellular matrix proteolysis^8^, and the amoeboid mode, in which carcinoma cells with low adhesion and round morphology require subcellular localization of myosin II behind the cell nucleus to drive actomyosin contractility, independently of matrix metalloproteinase^9^,^10^,^11^. Moreover, mesenchymal cells usually burst after entering the bloodstream, whereas highly metastatic cancer cells efficiently penetrate the blood vessels through amoeboid-like migration with high actomyosin contractility, which provides cells with mechanical strength to resist shear forces in the circulation^9^,^10^,^12^. In addition, cells undergoing amoeboid migration have higher velocity than those undergoing mesenchymal migration^13^. Despite this observation, the molecular mechanisms that affect the amoeboid motility in a 3D environment require more in-depth study.

Vinculin (*VCL*), a membrane cytoskeletal protein found in focal adhesion plaques, is involved in the linkage of the ECM to the actin cytoskeleton^14^. Vinculin-null cells transfected with vinculin cDNA show markedly decreased motility and tumorigenicity^15^. In addition, downregulation of vinculin was found in several highly metastatic cancer cells^16^,^17^. Loss of vinculin induced protection from apoptosis in an anchorage-independent manner and enhanced cell motility^18^. However, few studies have described the involvement of vinculin in the regulation of breast cancer amoeboid movement.

In this study, we show that loss of ERα promotes tumour metastasis through *in vitro* experiments, *in vivo* tumour xenograft assays and the analysis of clinical breast cancer samples. Furthermore, we find that ERα is a novel regulator of vinculin expression in breast cancer and that loss of ERα induces amoeboid-like migration of breast cancer cells by regulating vinculin in a 3D matrix.

## Results

### Loss of ERα is correlated with breast cancer metastasis

**Figure 1:**
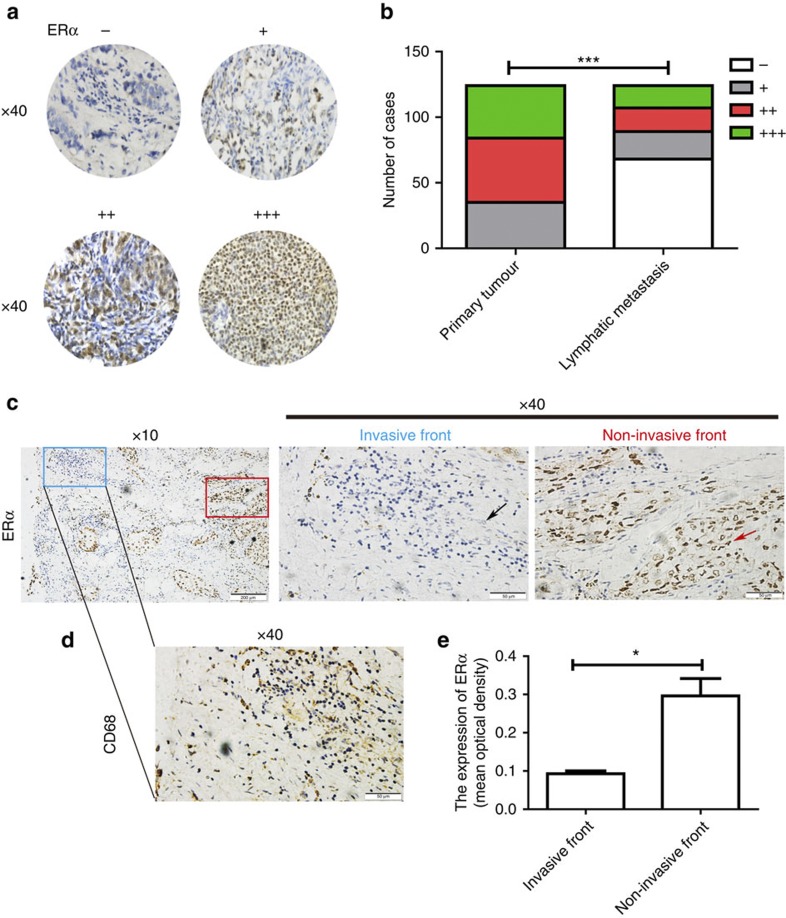
Loss of ERα is significantly correlated with breast cancer metastasis. Immunohistochemistry was performed using a specific antibody against ERα. (**a**) Representative images of ERα expression levels. (**b**) The expression level of ERα was significantly lower in lymph node metastasis than in the primary tumour. (**c**) Representative immunohistochemical staining for ERα at the invasive front (blue) and the non-invasive front (red) of human breast cancer. Scale bars, 200 μm (× 10) and 50 μm (× 40). The black arrow indicates breast cancer cells at the invasive front and the red arrow indicates breast cancer cells at the non-invasive front. (**d**) A serial section was used for immunohistochemical staining of CD68 at the invasive front of **c**. Scale bar, 50 μm (× 40). (**e**) The mean optical density of ERα expression at the invasive front was lower than that at the non-invasive front. Three invasive fronts and non-invasive fronts from **c** were analysed. Graph shows mean±s.e.m. *P<0.05, ***P<0.001. (**b**) Wilcoxon rank-sum test; (**e**) unpaired *t*-test.

Metastasis occurs when tumour cells detach from their primary location and move to the lymph nodes and then to distant organs^19^,^20^. Therefore, the lymph node usually serves as a bridge allowing the metastatic dissemination of tumours^21^. We examined ERα expression in human primary breast cancer tissues and the corresponding lymph node metastasis from 124 ERα-positive breast cancer patients. This analysis demonstrated that, in contrast to the abundant expression of ERα in the primary tumour, 54.8% of samples lost the expression of ERα in the corresponding lymphatic metastasis (Fig. 1a,b) (Supplementary Table 1). In addition, loss of ERα expression in lymphatic metastasis was also positively associated with the clinical stage (*P*=0.007), the number of lymph node metastases (*P*=0.011) and the loss of progesterone receptor expression (*P*=0.045). However, there was no significant association with age, tumour size or HER2 expression (Supplementary Table 2).

We further examined the expression of ERα at the invasive front where the infiltration of CD68-positive tumour-associated macrophages is found^22^ and where at least 50% of the cell surface of tumour cells contacts the matrix^23^ and the non-invasive front of the primary tumour tissues. The results showed that ERα was minimally expressed in breast cancer cells at the invasive front, whereas an increased intensity of ERα staining was observed at the non-invasive front (Fig. 1c–e). Collectively, these findings suggest that decreased ERα expression might promote breast cancer metastasis.

### ERα inhibits breast cancer metastasis *in vivo* and *in vitro*

**Figure 2:**
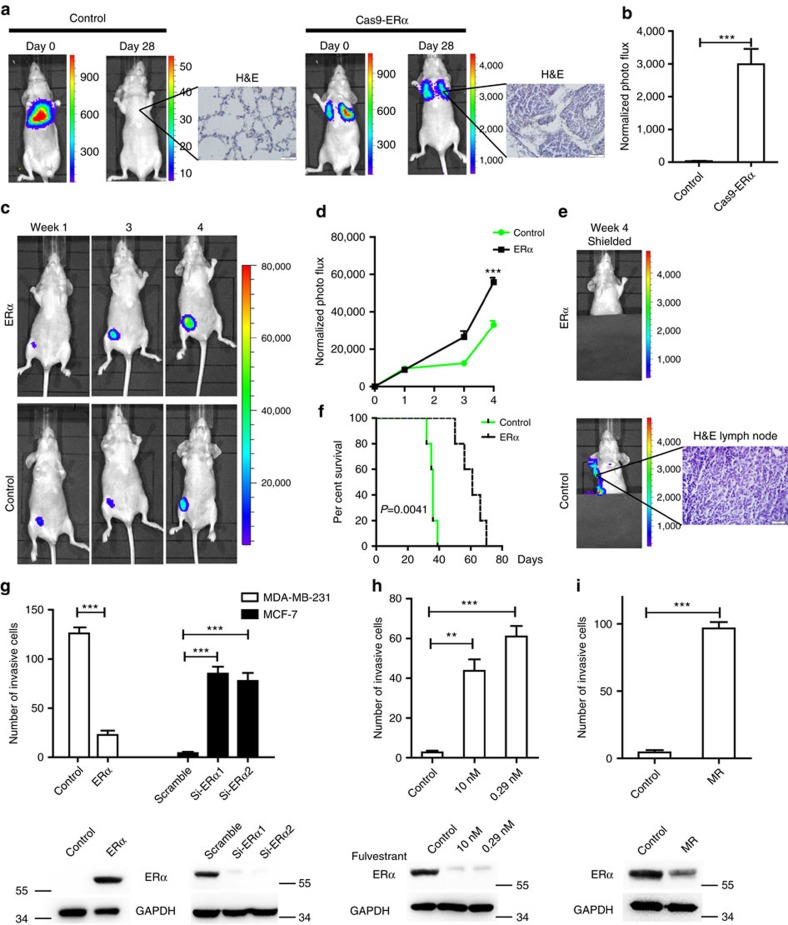
ERα inhibits breast cancer metastasis *in vivo* and *in vitro*. MCF-7-luc2 (control) or CRISPR/Cas9-mediated *ESR1*-deleted MCF-7-luc2 cells (Cas9-ERα) were injected via tail veins into nude mice (*n*=5). (**a**) Bioluminescence imaging of the control or the Cas9-ERα group at different time points was used to evaluate tumour progression in the lung. The lung metastases were determined using H&E staining. (**b**) Luciferase counts of the metastasis sites of mice on week 4. MDA-MB-231-luc2 (control) or ERα-overexpressing MDA-MB-231-luc2 (ERα) cells were injected into nude mice to generate xenograft models (*n*=5). (**c**) Bioluminescence imaging at different time points was used to evaluate tumour progression. (**d**) Luciferase counts of the primary tumours of mice at different time points. (**e**) Representative images of mice on week 4 are shown after shielding the primary tumour. The lymphatic metastases were determined with H&E staining. (**f**) The lifetimes of mice injected with control or ERα-overexpressing cells. (**g**) A transwell assay (top) was performed to determine the effect of ERα on cell invasion by gain or loss of ERα in MDA-MB-231 or MCF-7 cells (*n*=3). The expression of ERα (bottom) in different groups of MDA-MB-231 or MCF-7 cells was detected by western blotting. (**h**) A transwell assay (top) was performed to determine the effect of fulvestrant on the invasive capability of MCF-7 cells (*n*=3). The expression of ERα (bottom) in MCF-7 cells that were processed with fulvestrant of different concentrations was detected by western blotting. (**i**) A transwell assay (top) was performed to determine the invasive capability of parental MCF-7 and MR cells (*n*=3). The expression of ERα (bottom) was detected by western blotting. (**b**,**d**,**g**–**i**) Graphs show mean±s.e.m. **P<0.01,***P<0.001. (**b**,**d**,**g**,**i**) Unpaired *t*-test; (**f**) log-rank test; (**g**,**h**) analysis of variance (ANOVA) with Dunnett *t*-test.

To investigate whether ERα might inhibit the metastasis of breast cancer cells *in vivo*, we first chose ERα-positive MCF-7 and Cas9-ERα MCF-7 cells to examine the lung metastasis in a tail vein injection model, which mimics the process of loss of ERα during metastasis in patients. The expression of ERα in the two cell lines was detected by western blot analysis (Supplementary Fig. 1a,b). The cell proliferation assay confirmed that the Cas9-ERα MCF-7 cells grew more slowly *in vitro* (Supplementary Fig. 1c). Biofluorescence was examined at different time points to monitor the location and growth of tumour xenografts in the lungs. We found that the normalized photon flux of Cas9-ERα MCF-7 cells, which represents the pulmonary metastasis focuses, was significantly higher than that of control cells, as evidenced by H&E staining (Fig. 2a,b). Therefore, the Cas9-ERα MCF-7 cells showed a more profound increase in metastatic potential to the lungs.

Furthermore, control MDA-MB-231 cells and ERα-expressing MDA-MB-231 cells were also used to examine the metastasis in an orthotopic injection model (Fig. 2c). The expression levels of ERα in these two cell lines were quantified *in vitro* (Supplementary Fig. 1d,e). The normalized photon flux of control cells was lower than that of ERα-expressing cells (Fig. 2d), and the cell proliferation assay also verified that the control cells grew more slowly *in vitro* (Supplementary Fig. 1f). To observe the tumour metastasis, we covered the primary tumour site to avoid strong signal interference from the primary tumour. We found that the tumours derived from control cells were more metastatic than the tumours from ERα-expressing cells (Fig. 2e; Supplementary Fig. 1g). Further histological examination of the ipsilateral side of axillary lymph nodes showed lymphatic metastases in mice inoculated with control cells (Fig. 2e). Because metastasis accounts for ∼90% of breast cancer-related deaths^24^,^25^, we recorded the survival rate of nude mice in two additional groups. The results showed that the overexpression of ERα significantly extended the lifespan of the mice (Fig. 2f).

Transwell assays were performed with MDA-MB-231 and MCF-7 cells with gain or loss of ERα, and the results showed that ERα inhibited breast cancer cell invasion *in vitro* (Supplementary Fig. 1h–j; Fig. 2g). We also conducted transwell assays with MCF-7 cells that were treated with different concentrations of fulvestrant, an ERα downregulator. The results showed that, although 0.29 and 10 nm fulvestrant significantly downregulated ERα expression and inhibited cell proliferation, it promoted cell invasion (Fig. 2h; Supplementary Fig. 1k–m). In addition, we found that the invasive capacity of tamoxifen-resistant MCF-7 (MR) cells with lower ERα expression was significantly stronger than that in parental MCF-7 cells (Fig. 2i; Supplementary Fig. 1n,o). These observations indicated that loss of ERα expression promotes the invasive and metastatic ability of breast cancer cells.

### Loss of ERα induces the amoeboid migration of MCF-7 cells

**Figure 3:**
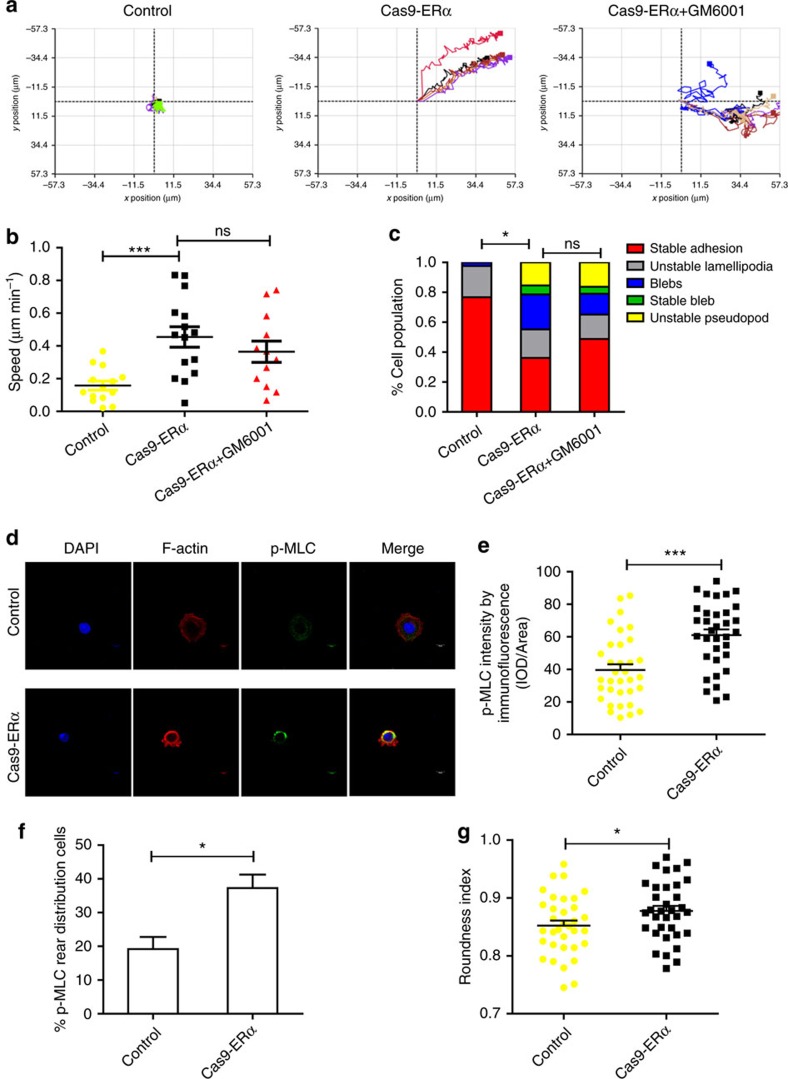
Loss of ERα induces the amoeboid migration of MCF-7 cells. (**a**) Representative tracks of control MCF-7 cells, Cas9-ERα MCF-7 cells and GM6001-treated Cas9-ERα MCF-7 cells. (**b**) The migration speed of control MCF-7 cells, Cas9-ERα MCF-7 cells and GM6001-treated Cas9-ERα MCF-7 cells in the 3D matrix (*n*=14, 15, 12 cells, respectively). (**c**) The percentage of cells exhibiting different phenotypes in the 3D matrix for control MCF-7 cells, Cas9-ERα MCF-7 cells and GM6001-treated Cas9-ERα MCF-7 cells (*n*=43, 47, 43 cells, respectively). (**d**) Representative confocal images of p-MLC and F-actin immunostaining of control and Cas9-ERα MCF-7 cells that were embedded in Matrigel. Scale bars, 10 μm. (**e**) Quantification of p-MLC expression levels from confocal images of **d** (*n*=34 cells). (**f**) The percentage of cells exhibiting p-MLC rear distribution for control MCF-7 cells and Cas9-ERα MCF-7 cells (*n*=3). (**g**) Cell morphology (roundness index) of control MCF-7 cells and Cas9-ERα MCF-7 cells (*n*=34 cells). (**b**,**e**–**g**) The data are shown as the mean±s.e.m. ns *P*>0.05, *P<0.05, ***P<0.001 (**b**) ANOVA with Tukey's *post hoc* test; (**c**) *χ*^2^-test; (**e**,**g**) unpaired *t*-test; (**f**) Wilcoxon rank-sum test.

The migration of invasive cells in the 3D environment consists of the mesenchymal mode and the amoeboid mode. Next, we tracked and analysed the migration of different MCF-7 cell populations of control, Cas9-ERα and GM6001 (matrix metalloproteinase inhibitor)-treated Cas9-ERα in the 3D matrix by using phase holographic imaging assays. The result showed that loss of ERα in MCF-7 cells promoted the invasive ability of tumour cells in the 3D matrix and that GM6001 did not impair the migration speed of Cas9-ERα cells (Fig. 3a,b). In addition, GM6001 did not affect the migration speed of MCF-7 cells either (Supplementary Fig. 2a,b). We also found that the control and Cas9-ERα MCF-7 cells did not express active MMP-2 or MMP-9 enzymes by zymography assay (Supplementary Fig. 2c). Using phase holographic imaging assays, we observed the following five phenotypes in the different populations of MCF-7 cells: ‘stable adhesion', ‘unstable lamellipodia', ‘bleb', ‘stable bleb' and ‘unstable pseudopod' (Supplementary Movies 1–5). The ‘bleb', ‘unstable pseudopod' and ‘stable bleb' phenotypes are known as amoeboid-like protrusions, which are associated with enhanced amoeboid movement^26^,^27^. The incidence of a ‘stable adhesion' phenotype was significantly reduced, and the amoeboid-like protrusions were more predominant in Cas9-ERα- or GM6001-treated Cas9-ERα cells compared with the control (Fig. 3c). Amoeboid migration requires high levels of phosphorylation of myosin light chain (p-MLC) and subcellular localization of p-MLC behind the cell nucleus to drive actomyosin contractility^11^,^23^. Confocal assays showed an increased expression of the active form (phospho-Ser19) of myosin light chain (p-MLC) in Cas9-ERα MCF-7 cells, and more Cas9-ERα MCF-7 cells showed p-MLC rear distribution (Fig. 3d–f). In addition, the roundness index of Cas9-ERα cells was higher than that of control cells (Fig. 3g). Together, these results indicated that loss of ERα induces the amoeboid-like migration of MCF-7 cells.

### ERα is a transcriptional promoter of vinculin

**Figure 4:**
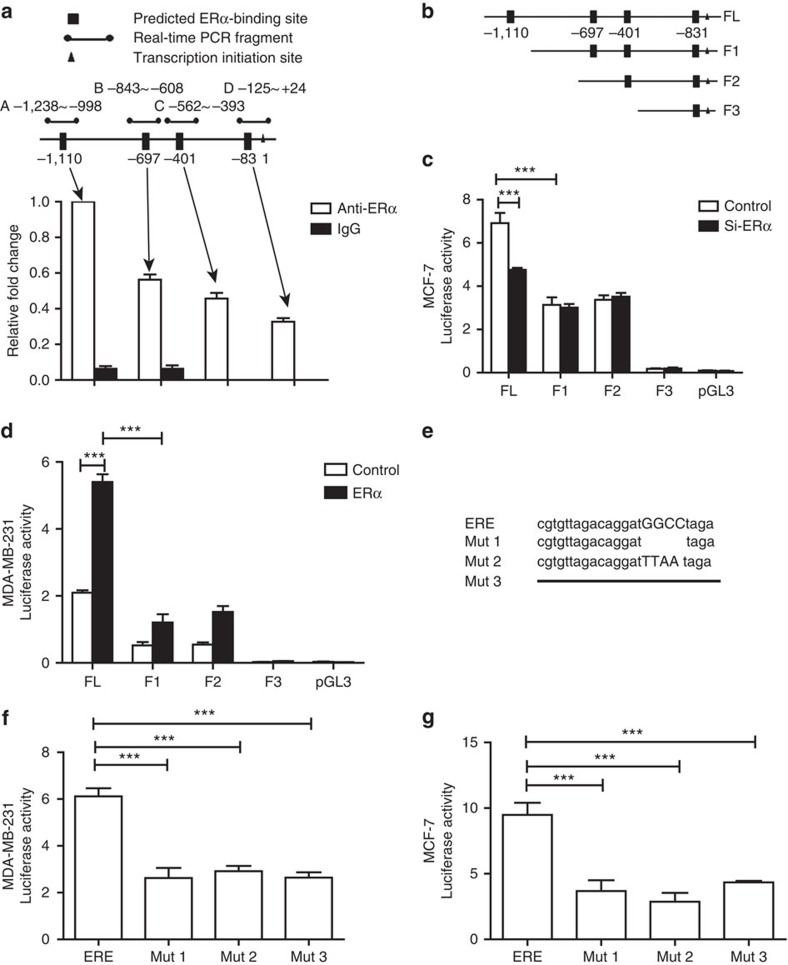
ERα is a transcriptional promoter of vinculin. (**a**) The schematic diagrams of human vinculin promoter containing four putative ERα-binding sites and the amplification regions of ChIP primers are presented in the top panel. The bottom panel shows the amounts of DNA fragments that were normalized to the total input genomic DNA from MCF-7 cells precipitated by either anti-ERα monoclonal antibody or control IgG (*n*=3). (**b**) Schematic diagrams of vinculin promoter truncation. FL indicates the full length of the vinculin promoter, and the corresponding truncation is represented by F1, F2 and F3. (**c**) Luciferase activity was measured in MCF-7 cells co-transfected with truncated vinculin promoter and ERα siRNA. pGL3-basic plasmid was used as a negative control (*n*=3). (**d**) Luciferase activity was measured in MDA-MB-231 cells co-transfected with the truncation of the vinculin promoter and the ERα vector (*n*=3). (**e**) Schematic diagrams of a 23-bp ERE in the vinculin promoter and respective mutation. Mut1: deletion of GGCC sequence; Mut2: replacing GGCC with TTAA; Mut3: totally deleting ERE. (**f**) Luciferase activity was measured in MDA-MB-231 cells transfected with mutations of the vinculin promoter and ERα vector (*n*=3). (**g**) Luciferase activity was measured in MCF-7 cells transfected with mutations of the vinculin promoter (*n*=3). (**a**,**c**,**d**,**f**,**g**) The data are shown as the mean±s.e.m. ***P<0.001 (**a**,**c**,**d**,**f**,**g**) ANOVA with Dunnett *t*-test.

To elucidate the molecular mechanism of ERα action on the metastasis of breast cancer, RNA-sequencing was performed using MDA-MB-231-ERα cells and MDA-MB-231-vector cells. The main metastasis-associated genes that showed significantly altered expression levels are shown in Supplementary Table 3. The vinculin transcript was most significantly altered, and this result was verified by real-time PCR (Supplementary Fig. 3a). Using bioinformatics analysis, we found four potential ERα-binding sites in the promoter of vinculin by which ERα might regulate vinculin transcription. Subsequently, MCF-7 cells cultured in oestrogen-depleted medium were treated with 0 or 5 nm oestrogen, and the luciferase activity of the vinculin promoter was determined in these MCF-7 cells. We found that, when the cells were treated with oestrogen, the luciferase activity increased significantly (Supplementary Fig. 3b). Next, we performed chromatin immunoprecipitation (ChIP) assays in MCF-7 cells to detect whether endogenous ERα formed a transcription initiation complex at the vinculin promoter. From quantitative PCR analysis, we found that the antibody against ERα pulled down higher amounts of vinculin promoter DNA than did the IgG control and that the most efficient binding activity was localized within the −998 to −1,238 region of the vinculin promoter (Fig. 4a).

Then, we truncated the vinculin promoter according to the four ERα candidate-binding sites, as shown in Fig. 4b, seeking to identify which region of the promoter is important for vinculin transcription by using a dual luciferase reporter assay. The results showed that the essential region for ERα transcriptional activity in the promoter was −940 to −1,155 bp in both MCF-7 and MDA-MB-231 cells (Fig. 4c,d). Sequence analysis of this region revealed a 23-bp ERE located in the −940 to −1,155 bp region whose core sequence was GGCC (Fig. 4e). To verify the effect of the ERE region, three mutants were constructed, as shown in Fig. 4e and used in luciferase reporter assays. The results showed that all three mutants exhibited decreased luciferase activities in MDA-MB-231 and MCF-7 cells (Fig. 4f,g). Collectively, these results demonstrated that ERα directly facilitates vinculin transcription by specifically binding to the ERE region of the vinculin promoter.

### ERα upregulates vinculin expression in breast cancer cells

**Figure 5:**
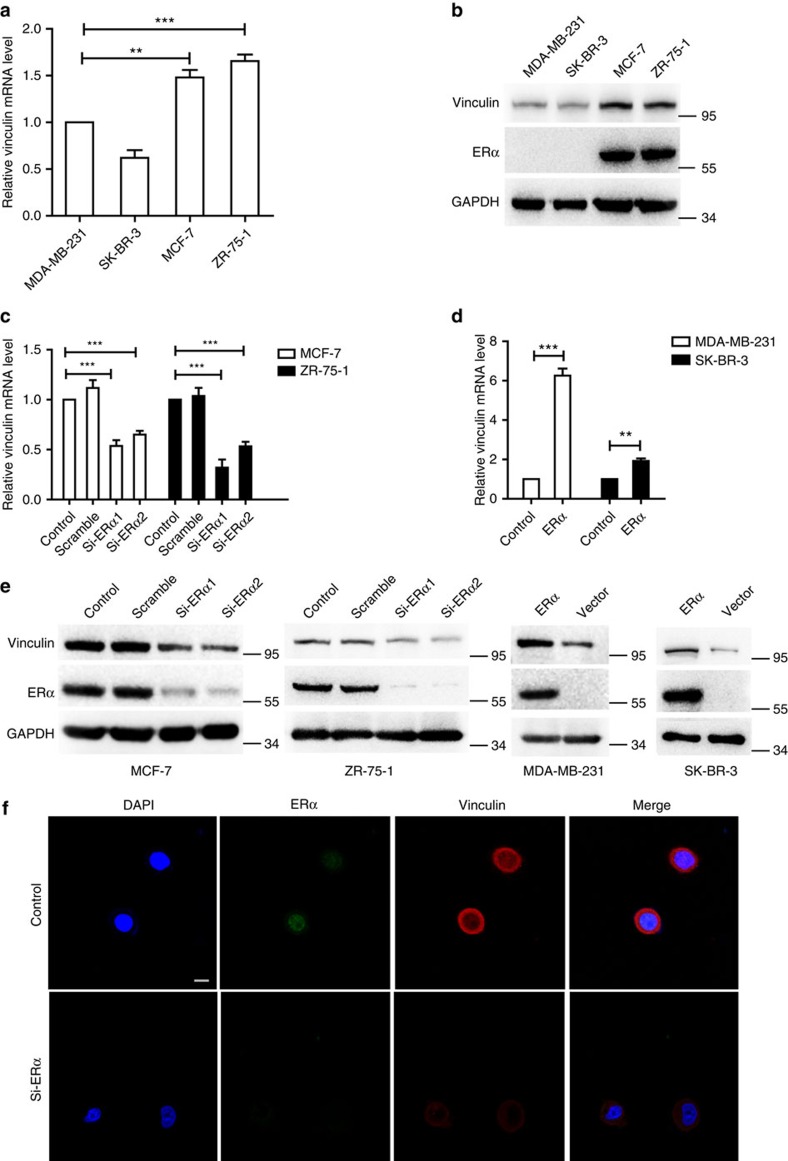
ERα upregulates the expression of vinculin in breast cancer cells. (**a**) Real-time PCR detecting the transcription levels of vinculin in breast cancer cell lines. The results were normalized to GAPDH (*n*=3). (**b**) Western blotting was conducted to detect the protein levels of vinculin and ERα in four breast cancer cell lines. (**c**) MCF-7 and ZR-75-1 cells were transfected with ERα siRNAs or scrambled RNA and subjected to quantitative reverse transcriptase PCR (qRT–PCR) assay (*n*=3). (**d**) MDA-MB-231 and SK-BR-3 cells that were transfected with ERα vector or vector subjected to qRT–PCR assay (*n*=3). (**e**) Western blotting to detect the protein level of vinculin and ERα protein by the gain or loss of ERα in breast cancer cell lines. (**f**) Confocal assay for ERα and vinculin expression in MCF-7 (control or si-ERα) cells. Nuclear staining with 4,6-diamidino-2-phenylindole (DAPI) is also shown. Scale bar, 10 μm. (**a**,**c**,**d**) The data are shown as the mean±s.e.m. **P<0.01,***P<0.001 (**a**,**c**) ANOVA with Dunnett *t*-test; (**d**) unpaired *t*-test.

MCF-7 cells cultured in oestrogen-depleted medium were treated with 0 or 5 nm oestrogen. We observed that the mRNA levels of vinculin were higher in MCF-7 cells treated with 5 nm oestrogen than in control cells (Supplementary Fig. 4a). In addition, the mRNA and protein levels of vinculin were higher in ERα-positive breast cancer cell lines than in ERα-negative cell lines (Fig. 5a,b; Supplementary Fig. 4b). To further investigate the role of ERα in regulating vinculin transcription and expression, real-time PCR and western blotting were performed to assess the effect of gain or loss of ERα expression on the level of vinculin mRNA and protein. The results showed that silencing endogenous ERα by using short interfering RNAs (siRNAs) led to a downregulation of vinculin in MCF-7 and ZR-75-1 cells and that ectopic expression of ERα by using the pcDNA3.1(−)-ERα vector in MDA-MB-231 and SK-BR-3 cells induced vinculin upregulation both at the mRNA and protein levels (Fig. 5c–e; Supplementary Fig. 4c–f). Importantly, using a confocal assay, we further confirmed that the expression of vinculin decreased after knockdown of nuclear expression of ERα in MCF-7 cells (Fig. 5f; Supplementary Fig. 4g). In addition, in MDA-MB-231 cells, vinculin expression was concomitantly promoted by the ectopic nuclear expression of ERα (Supplementary Fig. 4h). Together, these results indicated that ERα is a promoter of vinculin expression in breast cancer cells.

### Vinculin downstream of ERα is important for metastasis

**Figure 6:**
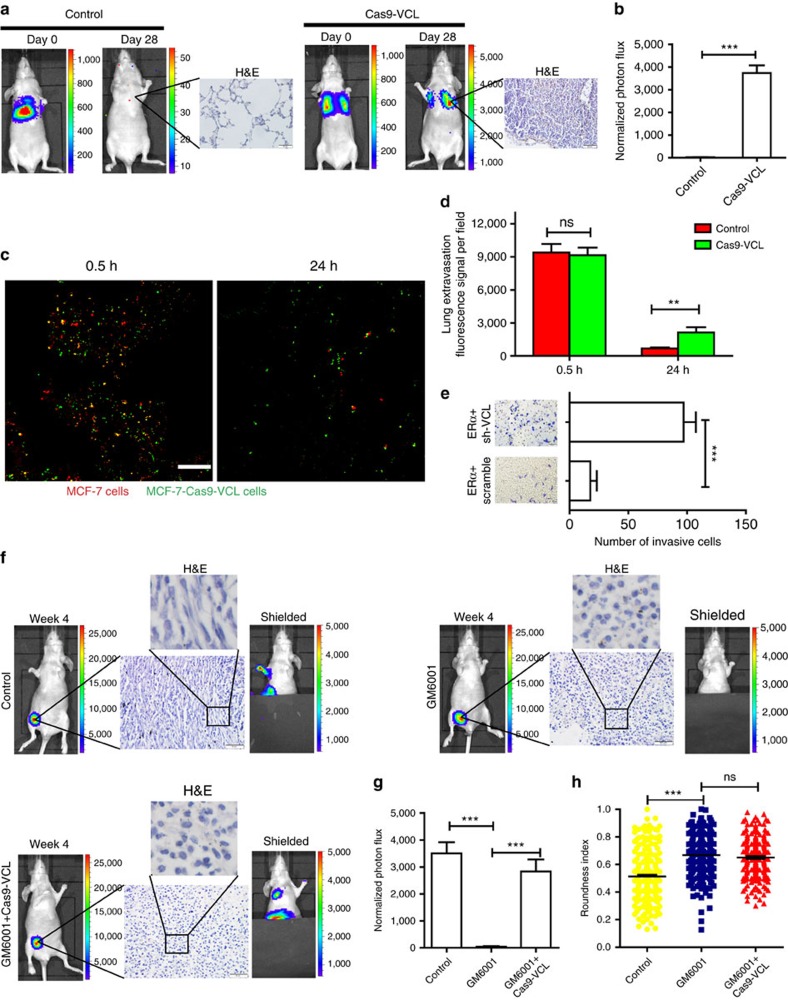
Vinculin downstream of ERα is important for metastasis. MCF-7-luc2 cells and CRISPR/Cas9-mediated *VCL*-deleted MCF-7-luc2 cells were injected into nude mice via the tail vein to generate xenografts (*n*=5). (**a**) Bioluminescence imaging of the control or the Cas9-vinculin group. Tumour formation in the lung was determined using H&E staining. (**b**) Luciferase counts in the lungs of mice on week 4. (**c**) Representative confocal images of mouse lungs 0.5 and 24 h after tail vein co-injection of control MCF-7 cells (red) and Cas9-vinculin MCF-7 cells (green). Scale bar, 100 μm. (**d**) Quantification of cells retained in the lung after tail vein injection (*n*=4 mice). (**e**) A transwell assay was performed in ERα-overexpressing MDA-MB-231 cells infected with lentivirus containing vinculin short hairpin RNA (shRNA; sh-VCL) or scrambled RNA (scramble; *n*=3). Control MDA-MB-231-luc2 cells, GM6001-treated control cells or GM6001-treated and CRISPR/Cas9-mediated *VCL*-deleted MDA-MB-231 cells were injected into nude mice to generate xenografts (*n*=5). (**f**) Representative bioluminescence images of different groups on week 4 are shown. The cell morphology of the primary tumour was determined by H&E staining. Scale bar, 50 μm. The particular section of H&E image was enlarged to highlight the cell morphology. (**g**) Luciferase counts of metastasis sites on week 4. (**h**) Roundness index of corresponding MDA-MB-231 cells from the primary tumour in **f** (*n*=200 cells). (**b**,**d**,**e**,**g**,**h**) The data are shown as the mean±s.e.m. ns *P*>0.05, **P<0.01, ***P<0.001. (**b**,**d**,**e**) Unpaired *t*-test; (**g**,**h**) ANOVA with Tukey's *post hoc* test.

To investigate the role of vinculin, downstream of ERα, in breast cancer metastasis, we performed tail vein injections of control or Cas9-vinculin MCF-7-luc2 cells in athymic mice and examined the expression levels of vinculin by western blotting (Supplementary Fig. 5a,b). We found that, compared with control cells, the Cas9-vinculin cells showed a more profound metastatic potential to the lungs (Fig. 6a,b). In addition, extravasation is a crucial step during tumour metastasis. To test whether vinculin involved in this process, the lung extravasation assay was performed. Similar numbers of control or vinculin-depleted MCF-7 cells lodged in the lung capillaries 0.5 h after injection (Fig. 6c). However, after 24 h the number of vinculin-depleted MCF-7 cells that remained in the lung parenchyma was more than the number of control cells (Fig. 6c,d).

We further studied an orthotopic mouse model of breast cancer, using MDA-MB-231 cells stably expressing ERα with or without vinculin knockdown (Supplementary Fig. 5c). Vinculin expression levels were detected by western blotting (Supplementary Fig. 5d,e). The results showed that tumours formed by vinculin knockdown cells were more metastatic compared with those of the control cells, and, after 4 weeks, lymph node metastasis was observed in the mice injected with vinculin knockdown cells (Supplementary Fig. 5f,g). The mice inoculated with vinculin knockdown cells had shorter lifetimes, probably because of the distant metastasis (Supplementary Fig. 5h). In addition, a transwell assay also showed that vinculin knockdown in MDA-MB-231 cells stably expressing ERα rescued the invasive capacity of the cells (Fig. 6e).

Furthermore, it has been reported that MDA-MB-231 cells can migrate through either the mesenchymal or amoeboid mode, and the amoeboid morphology might be predominant after protease inhibition^28^. Using zymography on conditioned two-dimensional (2D) or 3D medium, we observed that MDA-MB-231 cells expressed active MMP-2 or MMP-9 enzymes (Supplementary Fig. 5i). We also observed that GM6001 indeed decreased the invasive capacity of MDA-MB-231 cells in transwell assays (Supplementary Fig. 5j,k). Moreover, we observed that GM6001 induced round cell morphology and that depleted vinculin expression reversed the decreased invasive capacity of the GM6001-treated MDA-MB-231 cells to some degree (Supplementary Fig. 5j,k). Next, we performed orthotopic injection of control, GM6001-treated control or GM6001-treated Cas9-vinculin MDA-MB-231 cells in athymic mice. Tumours from GM6001-treated cells were less metastatic than those from control cells, and tumours from GM6001-treated Cas9-vinculin cells and control cells had similar levels of metastasis (Fig. 6f,g). In addition, GM6001-treated cells in primary tumours showed round cell morphology with a higher average roundness score than control cells *in vivo* (Fig. 6h). In addition, the mice inoculated with control or GM6001-treated Cas9-vinculin cells had shorter lifetimes than those inoculated with GM6001-treated control cells (Supplementary Fig. 5l). These results showed that GM6001 can induce the rounded-amoeboid morphology of cells and loss of vinculin in GM6001-treated breast cancer cells is associated with increased metastatic potential.

### Loss of vinculin promotes amoeboid features of cancer cells

**Figure 7:**
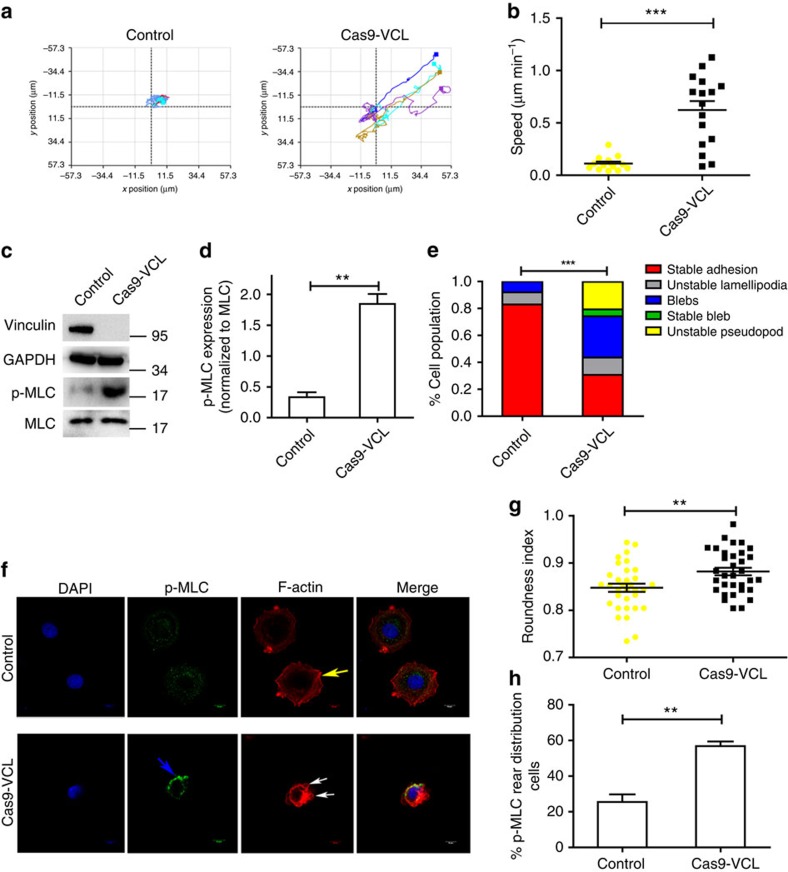
Loss of vinculin promotes amoeboid features of cancer cells. (**a**) Representative tracks of control and Cas9-vinculin MCF-7 cells. (**b**) The migration speed of control and Cas9-vinculin MCF-7 cells in the 3D matrix (*n*=14, 16 cells). (**c**) Western blotting to detect the expression levels of MLC and p-MLC. (**d**) Quantification of p-MLC expression in **c** normalized to total MLC (*n*=3). (**e**) The percentage of cells exhibiting different phenotypes in the 3D matrix for control and Cas9-vinculin MCF-7 cells (*n*=43 and 39 cells, respectively). (**f**) Representative confocal images of p-MLC and F-actin immunostaining of control and Cas9-vinculin MCF-7 cells that were embedded in Matrigel. Scale bars, 10 μm. The blue arrow indicates the accumulation of p-MLC at the cell rear; the white arrows indicate dynamic blebs; and the yellow arrow indicates stress fibre. (**g**) Cell morphology (roundness index) of control MCF-7 cells and Cas9-vinculin MCF-7 cells (*n*=33 cells). (**h**) The percentage of cells from **f** exhibiting p-MLC rear distribution for control and Cas9-vinculin MCF-7 cells (*n*=3). (**b**,**d**,**g**,**h**) The data are shown as the mean±s.e.m. **P<0.01, ***P<0.001. (**b**,**d**,**g**) Unpaired *t*-test; (**e**) *χ*^2^-test; (**h**) Wilcoxon rank-sum test.

The amoeboid features contained membrane blebbing, cell rounding, high actomyosin contractility and increased invasion^29^. To investigate whether loss of vinculin could promote the amoeboid features of cells, we tracked and analysed the migration speed of different MCF-7 cell populations of control and Cas9-vinculin in the 3D matrix by using phase holographic imaging assays (Fig. 7a). We found that loss of vinculin in MCF-7 cells promoted the instantaneous speed of tumour cells in the 3D matrix (Fig. 7b). In addition, after depletion of vinculin, the activity of MLC increased, thus indicating that loss of vinculin promoted actomyosin contractility in breast cancer cells (Fig. 7c,d). We also found that the percentage of amoeboid-like protrusions, including ‘bleb', ‘stable bleb' and ‘unstable pseudopod', in Cas9-vinculin cells significantly increased (Fig. 7e; Supplementary Movies 6–10). Confocal assays further showed that the control MCF-7 cells formed actin stress fibres, which were concomitant with p-MLC being evenly distributed in the cytoplasm, whereas in vinculin-depleted MCF-7 cells, few stress fibres were observed, and p-MLC was mostly polarized to the rear part of the cell (Fig. 7f). Moreover, Cas9-vinculin cells exhibited cell rounding and dynamic blebbing (Fig. 7f,g) and, compared with control cells, more Cas9-vinculin cells displayed p-MLC rear distribution (Fig. 7h).

### ERα correlates with vinculin in breast cancer tissues

**Figure 8:**
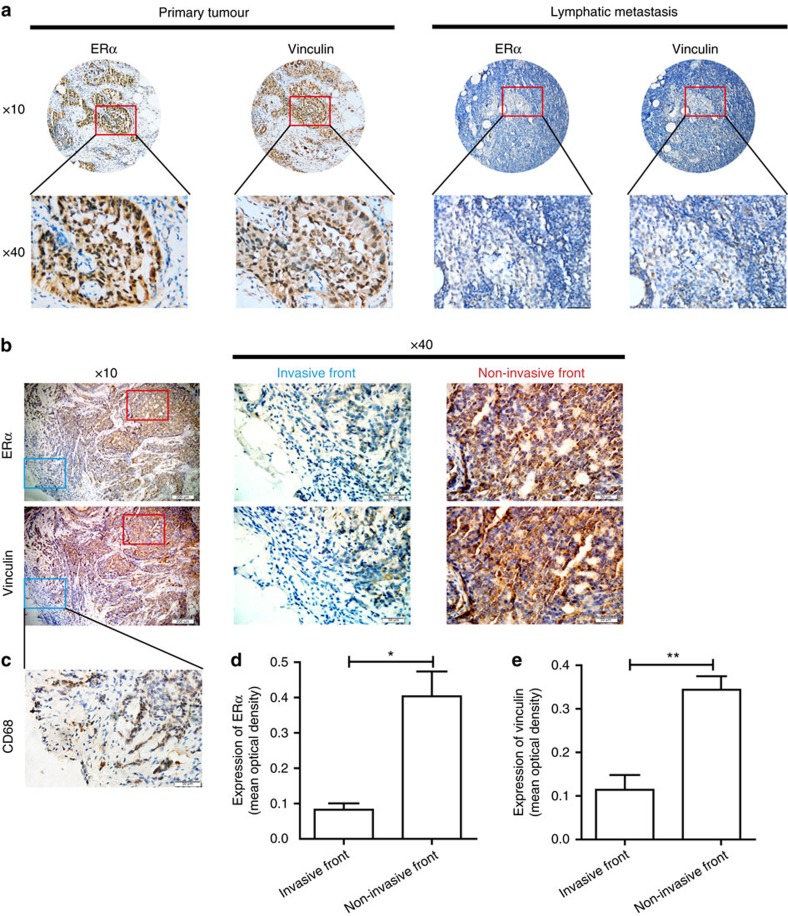
The ERα expression level in breast cancer tissues is positively correlated with the vinculin expression level in breast cancer tissues in a clinical setting. Immunohistochemistry analysis for determination of ERα and vinculin expression. (**a**) Representative images of ERα and vinculin expression in the primary tumour and corresponding lymphatic metastasis specimens. Scale bar, 50 μm (× 40). (**b**) Representative immunohistochemical staining for ERα and vinculin at the invasive front (blue) and the non-invasive front (red) of the human breast cancer primary tumour. Scale bars, 200 μm (× 10) and 50 μm (× 40). (**c**) Representative immunohistochemical staining for CD68 at the invasive front of **b**. (**d**,**e**) The mean optical density of ERα and vinculin expression at the invasive front was lower than that at the non-invasive front. Three invasive fronts and non-invasive fronts from **b** were analysed. (**d**,**e**) The data are shown as the mean±s.e.m. *P<0.05, **P<0.01. (**d**,**e**) Unpaired *t*-test.

To investigate in a clinical setting whether ERα suppresses breast cancer metastasis by facilitating vinculin expression, we measured the expression of vinculin in the same 124 human primary breast cancer tissues and human breast cancer lymph node metastases that were used in the ERα analysis. The results showed that vinculin abundance was also significantly higher in human primary breast cancer tissues than in lymphatic metastases (Fig. 8a). A positive association was found between vinculin and ERα in both primary tumour (*P*<0.001, *R*^2^=0.528) and lymphatic metastasis (*P*<0.001, *R*^2^=0.366; Supplementary Tables 4 and 5). The expression levels of vinculin are shown in Supplementary Fig. 6a.

We further compared the expression of these two molecules at the invasive and non-invasive fronts in primary breast cancer tissue. Similarly to the trend of ERα expression level, diminished vinculin expression was also detected at the invasive front, whereas increased staining of vinculin was observed at the non-invasive front (Fig. 8b–e). These findings indicated that ERα might suppress human breast cancer metastasis by facilitating vinculin expression.

## Discussion

Among all breast cancers, ERα-positive (ER+) tumours constitute the largest proportion, ∼70% (ref. 30). Although sporadic publications have shown that the expression of ERα in MDA-MB-231 cells inhibits proliferation *in vitro*^31^, a plethora of laboratory and epidemiological data have demonstrated that ERα, by binding to oestrogen, is the major driving factor for growth in ERα+ breast cancers^4^,^32^. In the present study, we also found that expression of ERα indeed promoted proliferation of MDA-MB-231 cells *in vitro* and *in vivo*. ERα induces cell proliferation by increasing the expression of MYC and cyclin D1 (refs 33, 34). Therefore, ERα has been used as a key target for endocrine therapy of ERα-positive breast cancer to block the proliferation of cancer cells^35^.

However, an increasing number of the clinical epidemiological investigations show that ERα-positive primary breast cancer patients have an increased frequency of *ESR1* mutations in metastatic ERα+ breast cancer tissues and even ERα-negative metastatic relapse after receiving endocrine therapy^1^,^7^. Although a decline in ERα levels has been detected in invasive breast cancers^4^, the relationship between ERα and tumour metastasis is still far from clear. In the present study, we observed that, compared with those in the primary tumour, breast cancer cells in lymph node metastases expressed lower levels of ERα. More importantly, loss of ERα in lymphatic metastases was also positively associated with clinical stages and lymph node metastases. Even in the primary tumour, the expression of ERα at the invasive front was lower than that at the non-invasive front. Therefore, these findings indicated that ERα expression is inversely correlated with breast cancer metastasis. We further demonstrated that ERα is indeed capable of inhibiting breast cancer metastasis by using athymic mouse models and transwell assays. This finding is very important because it suggested that breast cancer cells with loss of expression of ERα should be not only be resistant to common adjuvant endocrine therapy but also have stronger invasion and metastasis capabilities. Consistently with this possibility, we found that an appropriate concentration of fulvestrant promoted cell invasion *in vitro* by downregulating ERα and that the invasive capacity of tamoxifen-resistant MCF-7 cells with lower ERα expression was stronger than that of parental MCF-7 cells. Collectively, we believe that repeated biopsies are necessary to reassess the receptor status in metastatic disease to guide endocrine therapy with greater precision.

Several studies have reported that ERα suppresses cellular motility and invasion^31^ by inhibiting the epithelial–mesenchymal transition in 2D conditions^36^,^37^,^38^,^39^. However, with regard to cell shape and movement, the 3D environment resembles the *in vivo* condition^40^. Some tumour cells can utilize amoeboid-like migration as an alternative to mesenchymal migration during migration in 3D cultures and *in vivo*^41^,^42^. To date, few studies have focused on the relationship between the amoeboid-like migration of breast cancer cells in a 3D environment and ERα expression. We demonstrated that ERα-positive MCF-7 cells expressed few active MMP-2 and MMP-9. Loss of ERα also promoted a more round shape and increased cell motility in the 3D matrix *in vitro*, and these effects were not impaired by the MMP inhibitor GM6001. The formation of amoeboid-like protrusions, such as blebs, often corresponds with the amoeboid phenotype and enhanced invasion and metastasis and blebbing movement has been widely promoted as a cancer cell migration strategy^43^,^44^,^45^,^46^,^47^. We observed the significantly increased formation of amoeboid-like protrusions in ERα-depleted MCF-7 cells in the present study. During the initiation of blebs, local dissociation of the membrane from the cortex or a local rupture of the actin cortex causes the bleb to form in the direction of the desired flow^48^,^49^. Furthermore, it has been reported that high contractility of the cell rear triggers bleb initiation^43^,^50^. Indeed, we observed that loss of ERα in MCF-7 cells induced the polarization of p-MLC at the cell rear, thereby resulting in high contractility of the cell rear, the formation of amoeboid-like protrusions and enhanced amoeboid-like migration. Even in the MDA-MB-231 cells that use mixed migration modes in a 3D matrix, ERα suppressed amoeboid-like migration after MMP inhibition, although overexpression of ERα did not alter the mesenchymal-like morphology of MDA-MB-231 cells in a 2D substrate (Supplementary Fig. 6b,c). Together, our results show that ERα inhibits the amoeboid-like migration of breast cancer cells in a 3D matrix.

With respect to the mechanisms by which ERα inhibits cancer matastasis, we focused on the regulation of ERα to vinculin expression based on transcriptome sequence analysis. Our data show that ERα is a novel regulator of vinculin expression in breast cancer and that vinculin is involved in ERα-mediated inhibition of breast cancer cell metastasis. As above, vinculin is a key regulator of focal adhesions and loss of vinculin in cells promotes focal adhesion turnover^51^. It was reported that focal adhesions could promote the formation of persistent stress fibres, which prevent the transition to amoeboid migration by competing with the cell cortex for recruitment of the actomyosin contractile machinery^52^. In the present study, our results showed that the depletion of vinculin in MCF-7 cells induced decreased cell–matrix adhesion (Supplementary Fig. 6d). More importantly, we found that in a 3D matrix vinculin depletion leads to decreased stress fibres and p-MLC mostly polarizes to the rear part of the cell, thus resulting in high contractility of the cell rear and induced bleb initiation.

Metastasis occurs when tumour cells detach from the epithelial sheets and invade surrounding tissue. It was reported that vinculin was important for cadherin-mediated cell–cell junctions^53^ and might suppress metastasis formation *in vivo* by promoting cadherin-mediated retention of tumour cells in primary tumours^16^. Meanwhile, we found that depletion of vinculin in MCF-7 cells induced loss of cell–cell contact (Supplementary Fig. 6e,f). However, we also found that loss of vinculin in MCF-7 cells induced cell rounding, increased actomyosin contractility, fast shape changes and increased invasion. These observations indicated that the effect of vinculin on amoeboid features of cells might contribute to the process of local invasion or even the metastasis formation besides the effect on cell–cell adhesion. Moreover, it was reported that high levels of actomyosin contractility in cancer cells could promote and efficient lung colonization or seeding^54^,^55^ and rapid extravasation was associated with the amoeboid features^10^,^43^,^56^. Our *in vivo* extravasation assay showed that the number of vinculin-depleted cells that extravasated into the lung parenchyma after 24 h was more than the number of control cells. These observations indicated a potential involvement of vinculin in efficient retention in the lungs via its regulation of amoeboid features. More importantly, we do not discard the possibility that reduced cell–cell adhesion and anoikis resistance that resulted from loss of vinculin may be involved in metastasis; however, we believe that we have identified amoeboid features regulated by vinculin, which are associated with metastasis.

In summary, our results demonstrate that ERα suppresses breast cancer metastasis by regulating vinculin. Our findings provide novel insight into the association of ERα loss during endocrine therapy with enhanced invasive and metastatic ability of breast cancer cells and should aid in a more comprehensive understanding of the effects of endocrine therapy in clinical treatment.

## Methods

### Antibodies and inhibitors

Antibodies and dilutions used were as follows: ERα (ab32063: immunohistochemistry, 1:150; immunoblotting, 1:750; immunofluorescence, 1:150) from Abcam; vinculin (ab18058: immunohistochemistry, 1:100; immunoblotting, 1:400; immunofluorescence, 1:150) from Abcam; CD68 (916104: immunohistochemistry, 1:100) from BioLegend; GAPDH (CW0101: immunoblotting, 1:1,000) from CWBIOTECH; p-MLC(3671: immunoblotting, 1:750; immunofluorescence, 1:50) from Cell Signaling Technology; MLC (10906-1-AP, immunoblotting, 1:500) from Proteintech; F-actin (40734ES75: immunofluorescence, 1:100) from YEASEN; fluorescein isothiocyanate (FITC)-conjugated anti-rabbit and Cy3-conjugated anti-mouse antibodies from ZHUANGZHIBIO.

The inhibitors are as follows: *in vivo*: GM6001 (MedChem Express, Monmouth Junction, NJ, USA) was subcutaneously injected every 3 days at 100 mg per kg body weight^57^. *In vitro*: GM6001 (27 nm).

### Cell lines and culture

Human breast cancer cell lines MCF-7, ZR-75-1, MDA-MB-231 and SK-BR-3 were obtained from the Type Culture Collection of the Chinese Academy of Sciences (Shanghai, China). The breast cancer cell line MDA-MB-231-1uc2, which expresses luciferase, was a kind gift from Dr Xia Haibin of Shaanxi Normal University^58^. The parental MCF-7 cells and tamoxifen-resistant MCF-7 cells were gifted from Dr Zhang of the Fourth Military Medical University^59^. All cell lines were authenticated by the analysis of short tandem repeat (STR) profiles and 100% matched the standard cell lines in the DSMZ data bank. All cells were tested negative for cross-contamination of other human cells and mycoplasma contamination.

The cells were cultured in a corresponding medium without phenol red and supplemented with 10% oestrogen-deprived fetal bovine serum (FBS; HyClone SH30068.03, South Logan, USA) and 100 μg per ml ampicillin/streptomycin. Twenty-four hours before the experiment, oestrogen (Sigma-Aldrich, St Louis, MD, USA) was added with the final concentration of 5 nm.

### Plasmid construction and RNA interference

The pcDNA3.1(−)-ERα plasmid was retained by our laboratory. The synthesized nucleotides encoding wild-type, truncated or mutant vinculin promoters were digested with Kpn1 and Xho1 and cloned into a pGL3-basic vector. Three ERα siRNA molecules (Gene Pharma, Shanghai, China) were used to knockdown ERα expression in breast cancer cells. The vinculin short hairpin RNAs were designed and synthesized by GENECHEM (Shanghai, China). Scrambled RNAs were used as a negative control for nonsequence-specific effects. All sequences are listed in Supplementary Tables 6 and 7. siRNAs and plasmids were transfected into cells using Lipofectamine 2000 (Invitrogen, Carlsbad, CA, USA) following the manufacturer's instructions. The plasmid GV392 (Lenti-Case9-sgRNA-puromysin) was purchased from GENECHEM.

### Clinical specimens and immunohistochemistry

Patients were staged and classified according to the American Joint Committee on Breast Cancer Staging and Classification criteria. Specimens from 124 ERα-positive breast cancer patients with lymph node metastasis were obtained from The Department of Pathology or The Department of Vascular and Endocrine Surgery, The First Affiliated Hospital to The Fourth Military Medical University (FMMU, Shaanxi, China). The informed consent was obtained from all patients, and the study protocol was approved by the Ethics Committee of The Fourth Military Medical University.

Serial sections (4 μm) of paraffin-embedded samples were conventionally dewaxed and hydrated with gradient ethanol. After the inactivation of endogenous peroxidase with 3% H_2_O_2_–methanol for 10 min, the sections were washed three times in PBS and blocked with goat serum for 20 min. Then, the sections were coated with primary antibodies and incubated in a wet box at 4 °C overnight. After the addition of PowerVision complex (ZSGB-BIO, SP-9001, sp-9002), tumour sections were incubated at 37 °C for 20 min followed by 3, 3′-diaminobenzidine (DAB) (ZSGB-BIO, ZLI-9031) colouring and wood grain re-dyeing. PBS instead of antibodies was used as a negative control. With respect to ERα and vinculin staining, the percentage of stained cells was categorized as follows: −, no staining; +, 1–25%; ++, 25–50%; and +++, >50% staining. In parallel, corresponding haematoxylin-eosin (H&E) staining was reviewed to confirm diagnosis by a pathologist using the immunohistochemistry preparations.

### CRISPR/Cas9 system

GENECHEM designed and cloned the corresponding single guide RNAs (sgRNAs) into GV392 plasmid. Lentivirus was used to deliver the corresponding GV392 plasmid into breast cancer cell lines. The sequence of relative sgRNA was listed in Supplementary Table 8. The respective locus was amplified using the primers (*ESR1* fwd: 5′-TTGTAATGCATATGAGCTCG-3′ rev: 5′-CTGCTGTCCAGGTACACCTC-3′ and *VCL* fwd: 5′-CTTGTCCAGGCAGCTCAGA-3′ rev: 5′-CACCACCTCTGCCACTGT-3′). Heterodimerization and digestion were performed with the Knockout and Mutation Detection Kit (GENECHEM) according to the manufacturer's instructions. Cleavage products were separated on a 2% agarose gel and stained with ethidium bromide. Images were captured with the SmartGel Image Analysis System (SAGECREATION). The corresponding results were listed in Supplementary Fig. 6g,h.

### Animal studies

Female athymic mice, 4-week of age, were selected. The animal study was performed in accordance with a protocol approved by the Institutional Animal Care and Use Committee of the FMMU. For mammary fat pad injection, 3 × 10^6^ viable breast cancer cells were injected into the mammary fat pads of athymic mice (*n*=5). For tail intravenous injection, 1 × 10^6^ viable breast cancer cells were injected into athymic mice from tail vein (*n*=5). Then, the mice were anaesthetized and injected with luciferin and imaged using an IVIS-100 System at the UAB Small Animal Imaging Core Facility. Light emission from animal tissue was measured using a software provided by the vendor (Xenogen). In parallel, an equivalent number of tumour cells were inoculated into the same sites of other athymic mice to record their survival time. The operator who performed injection of tumour cells was blinded with the group allocation. At least two independent experiments were performed.

### Cell proliferation assay

The proliferation ability of the cells was measured by Cell Counting Kit-8 (CCK-8) solution (7sea biotech, Shanghai, China). Briefly, breast cancer cells were seeded on 96-well plates (Corning, USA) at a concentration of 2 × 10^4^ cells per well and incubated at 37 °C overnight. The Cell Counting Kit-8 reagents were then added to a subset of wells when cells grew for 24, 48, 72 or 96 h. After the cells were incubated for 2 h at 37 °C, we quantified the absorbance at 450 nm using a microplate reader (Bio-Rad). Each group was made in quintuplicate.

### Zymography

The assessment of MMP-2 and MMP-9 activity was performed using a Gelatin Zymography Kit (Xin Sails Biotechnology, Shanghai, China). Briefly, conditioned serum-free medium of breast cancer cells grown on plastic for 36 h was loaded into the polyacrylamide gels containing 10% gelatin and then electrophoresed at 20 mA per gel for 90 min. Following electrophoresis, gels were rinsed twice with 10 ml 1 × Buffer A for 30 min at room temperature and incubated with 10 ml 1 × Buffer B for 3 h at 37 °C. Gels were then stained with 0.5% Coomassie Blue for 2 h and destained five times for 20 min with destaining solution (methanol:acetic acid:water=5:7:88). The protein molecular weight of MMP-2, MMP-9 and proMMP-9 is 66∼72, 92 and 130 kD.

### Phase holographic imaging assay

Overall, 3 × 10^5^ MCF-7 cells of different groups were embedded in Matrigel (diluted with serum-free medium) at 37 °C overnight. Half an hour before the experiment, the transwell insert containing Matrigel (diluted with 10% FBS) was seeded on 3D matrix as a chemoattractant, which was in the right side of the observation point. Then, HoloMonitorTM M4 (Phiab, Sweden) was used to track and record related parameters of the movement of cells in 3D matrix in 4 h. Motility and protrusion data were obtained using × 40 objective. The cells were imaged every 2 min and the movies were played back at 15 frames per second. The main criteria used to group cells into the different phenotypes were the size of protrusions (<2 μm or not) and the lifetime of protrusions (<2 min or not).

### Calculation of cell roundness

It was assessed by dividing the shortest diameter of each cell by the longest one (ratio-b/ratio-a) to produce a score between 0 and 1, with perfectly round cells having a score of 1 (ref. 9).

### ChIP assay

ChIP experiments were performed using the EZ ChIP Chromatin Immunoprecipitation Kit (Millipore, Billerica, USA). Four primer sets were designed to flank-related putative ERα-binding sites in the promoter region of vinculin. Details of the primer sequence are listed in Supplementary Table 9. Briefly, MCF-7 cells were fixed with 1% paraformaldehyde and sonicated seven times for 10 s each using a sonicator with a microtip in a 1.5-ml tube. Anti-ERα antibody or control human IgG was applied to pull down the chromatin associated with ERα. The chromatin–antibody complexes were collected with Protein G-Agarose. After washing and elution of the complexes from the beads, the DNA–protein crosslinks were reversed at 65 °C overnight. The amounts of the specific DNA fragment were then quantified by real-time PCR and normalized against the genomic DNA preparation from the same cells. Each group was made in triplicate.

### Luciferase reporter assay

Briefly, breast cancer MCF-7 or MDA-MB-231 cells were seeded in 24-well plates at 50% confluence and transfected with either ERα siRNAs or ERα plasmid using Lipofectamine 2000 and then co-transfected with pRL-Tk and vinculin promoter (pGL3-basic-vinculin). Thirty-six hours later, the cells were lysed in a passive lysis buffer (Promega, San Luis Obispo) and the luciferase activity was measured. Each group was made in triplicate.

### Quantitative real-time PCR

Total RNA was isolated from cultured cells with RNAiso Plus (Takara, Dalian, China), and cDNA was synthesized with the PrimeScript RT Reagent Kit (Takara). Then, 2 μl of cDNA was used for real-time PCR reactions in a Prism 7500 real-time thermocycler (Applied BioSystems, Foster City, CA, USA) with SYBR Green Ex Taq (Takara) according to the manufacturer's instructions. The primer sequences are provided in Supplementary Table 10. Each group was made in triplicate.

### Western blot analysis

In brief, proteins were transferred to polyvinylidene difluoride membranes after SDS–PAGE using a Bio-Rad Semi-Dry electrophoretic cell. Western blot analyses were performed using specific antibodies followed by an horseradish peroxidase (HRP)-conjugated IgG antibody. Enhanced chemiluminescence (Pierce) for HRP was used for immunoreactive protein visualization. Uncropped scans of the blots are shown in Supplementary Figs 7 and 8.

### Immunofluorescence analysis

For the observation of the subcellular localization of p-MLC and F-actin, 4 × 10^5^ breast cancer cells were embedded in Matrigel at 37 °C overnight. For the observation of the expression of ERα and vinculin, 4 × 10^5^ breast cancer cells were seeded on glass plates at 37 °C overnight. The cells were then washed twice with cold PBS and fixed with 4% paraformaldehyde for 20 min, followed by permeabilization with 0.2% Triton X-100 diluted with PBS for 30 min at room temperature. The cells were incubated overnight at 4 °C with corresponding antibody after being blocked for 30 min with goat serum. Then, the cells were incubated for 2 h with secondary FITC-conjugated or Cy3-conjugated antibodies. Nuclei were counterstained with 4,6-diamidino-2-phenylindole. Images were captured using a confocal microscope (FluoView FV1000, Olympus, Tokyo, Japan).

### Lung extravasation assay

Overall, 5 × 10^5^ Cas9-vinculin MCF-7 cells (enhanced green fluorescent protein, eGFP) and 5 × 10^5^ control MCF-7 cells (monomeric red fluorescent protein, mRFP) were mixed and injected into the tail vein of nude mice (female, 4 weeks old). Mice were killed after 0.5 or 24 h and the lungs were fixed (4% formaldehyde for 24 h) and examined for fluorescently labelled cells under a confocal microscope (FluoView FV1000, Olympus). Lung retention was represented as fluorescence signal (eGFP or mRFP from MCF-7 cells) per field. Each experiment had four mice per condition, and experiments were replicated three times.

### Transwell assay

A total of 3 × 10^5^ cells were placed into chambers coated with 100 μl of Matrigel (BD Biosciences, 356234). The chambers were then inserted into a 24-well plate and incubated for 24 h in a corresponding medium with 10% FBS before examination. The cells remaining on the upper surface of the membranes were removed, whereas the cells that migrated to the lower surface were fixed with 95% ethanol and stained in 4 g l^−1^ crystal violet solution. Finally, the invasive cells distributed in eight randomly selected views were counted under a microscope (× 20) and averaged.

### Cell adhesion assay

In all, 1 × 10^4^ cells were seeded on Matrigel-coated 96-well plate and cultured for 2 h. After washing, adhesion cells were counted using optical microscope (Olympus). The adhesion cells distributed in a randomly selected view were counted under a microscope (× 40). Each group was made in octuplicate.

### Binding of cells to recombinant E-cadherin

A 96-well plate was coated with 100 μl human recombinant E-cadherin Fc chimera (648-EC-100, R&D Systems)/well at 1.5 μg ml^−1^ in PBS at 37 °C for 1 h. The plate was washed with PBS 3 times and blocked by adding 100 μl per well 1% BSA in PBS at 37 °C for 30 min. Then 3 × 10^4^ cells per well are added to Recombinant Human E-Cadherin Fc Chimera coated plates and the plate was kept at 37 °C for 90 min. After washing, adhesion cells were counted by optical microscope (Olympus, Tokyo, Japan). The adhesion cells distributed in a randomly selected view were counted under a microscope (× 40). Each group was made in octuplicate.

### Statistical analysis

Statistical analysis was performed using the SPSS statistical software (SPSS16.0, Chicago, CA, USA). A value of *P*<0.05 was considered statistically significant. A random number table was used to randomize the mice into control and treatment groups. The numbers of mice (*in vivo*) were determined on the basis of our pre-tests and previous experience with similar experiments. Sample size was chosen to ensure adequate and statistically significant results. Investigators who determined the expression levels of ERα and vinculin and cell–cell adhesion were blinded with respect to the treatment allocation. The *in vitro* experiments were repeated at least three times. The statistical tests were two-sided.

### Data availability

The authors declare that the main data supporting the findings of this study are available within the article and its Supplementary Information Files. Extra data are available from the corresponding authors upon request.

## Additional information

**How to cite this article:** Gao, Y. *et al*. Loss of ERα induces amoeboid-like migration of breast cancer cells by downregulating vinculin. *Nat. Commun.* **8,** 14483 doi: 10.1038/ncomms14483 (2017).

**Publisher's note:** Springer Nature remains neutral with regard to jurisdictional claims in published maps and institutional affiliations.

## Supplementary Material

Supplementary Information Supplementary Figures and Supplementary Tables

Supplementary Movie 1 “Stable adhesion” was identified as an almost static phenotype that remained stable for about 60 min. Related to Figure 3.

Supplementary Movie 2 “Unstable lamellipodium” was characterized by a band that did not lead to a persistent protrusion but rather translocate as a “wave” along the cell perimeter. Unstable lamellipodium drove local protrusion in MCF-7 cells over 8 min time periods. Related to Figure 3.

Supplementary Movie 3 “Bleb” phenotype of MCF-7 cells in the 3D matrix, whose protrusions were short-lived (>2 min) and less than about 2 μm in size. Related to Figure 3.

Supplementary Movie 4 “Stable-bleb” phenotype of MCF-7 cells in the 3D matrix, which was identified as displaying an invariant polarized balloon-like shape. Related to Figure 3.

Supplementary Movie 5 “Unstable pseudopod” began in a small region of the cell periphery then drove a rapid extension of the cell periphery. The dynamic protrusions of “unstable pseudopod” were short-lived (>2 min) and relatively large to “blebs” (<2 μm). Related to Figure 3.

Supplementary Movie 6 “Stable adhesion” phenotype of MCF-7 cells in the 3D matrix. Related to Figure 7.

Supplementary Movie 7 “Unstable lamellipodium” phenotype of MCF-7 cells in the 3D matrix. Related to Figure 7.

Supplementary Movie 8 “Bleb” phenotype of MCF-7 cells in the 3D matrix. Related to Figure 7.

Supplementary Movie 9 “Stable-bleb” phenotype of MCF-7 cells in the 3D matrix. Related to Figure 7.

Supplementary Movie 10 “Unstable pseudopod” phenotype of MCF-7 cells in the 3D matrix. Related to Figure 7.
