# Supplementary material for: Loss of ERα induces amoeboid-like migration of breast cancer cells by downregulating vinculin
Source: Nat Commun. 2017 Mar 7;8:14483. doi: 10.1038/ncomms14483 (PMC5344302; doi:10.1038/ncomms14483)
Supplement: Supplementary Information — Supplementary Figures and Supplementary Tables [file ncomms14483-s1.pdf]

Supplementary Figures:

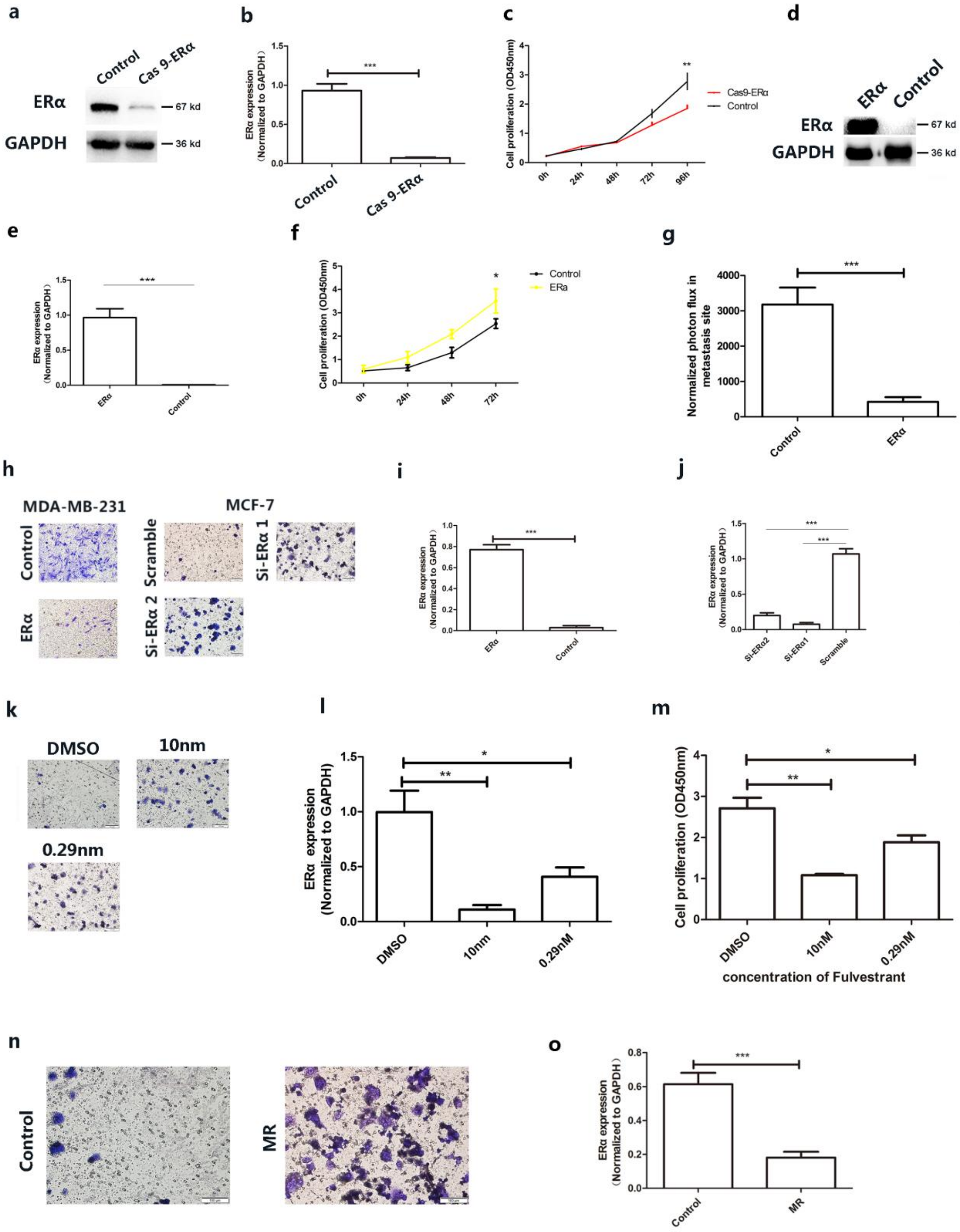

Supplementary Figure 1

Supplementary Figure 1: ERα inhibits breast cancer metastasis *in vivo* and *in vitro*. Related to figure 2. (**a**) Western blot assay for detecting the expression levels

of ERα in control and Cas9-ERα MCF-7 cells. (**b**) Quantification of ERα expression in control and Cas9-ERα MCF-7 cells normalized to GAPDH (n=3). (**c**) Cell proliferation assay for detecting the proliferative capability of control and Cas9-ERα MCF-7 cells (n=3). **(d)** Western blot assay for detecting the expression levels of ERα in MDA-MB-231-luc2-vector or MDA-MB-231-luc2-ERα cells. (**e**) Quantification of ERα expression in the two cell lines normalized to GAPDH (n=3). (**f**) Cell proliferation assay for detecting the proliferative capability of control and ERα-expressing MDA-MB-231 cells (n=3). (**g**) Luciferase counts at the metastasis sites of mice in Figure 2c at week 4. (**h**) A transwell assay was performed to determine the effect of ERα on cell invasion by gain or loss of ERα in MDA-MB-231 or MCF-7 cells. (**i, j**) Quantification of the ERα expression in MDA-MB-231 or MCF-7 cells from Figure 2g (n=3). (**k**) A transwell assay was performed to determine the effect of fulvestrant on the invasive capability of MCF-7 cells. (**l**) Quantification of ERα expression in MCF-7 cells treated with different concentrations of fulvestrant (n=3). (**m**) Cell proliferation assay (96 h) for detecting the proliferative capability of MCF-7 cells treated with different concentrations of fulvestrant (n=3). (**n**) A transwell assay was performed to determine the invasive capability of parental MCF-7 cells and MR cells. (**o**) Quantification of ERα expression in MCF-7 cells and MR cells (n=3). (**b, c, e, f, g, i, j, l, m, o**) Graphs show mean ± s.e.m. *P<0.05 **P<0.01***P<0.001. (**b, c, e, f, g, i, o**) Unpaired *t*-test; (**j, l, m**) ANOVA with Dunnett *t* test.

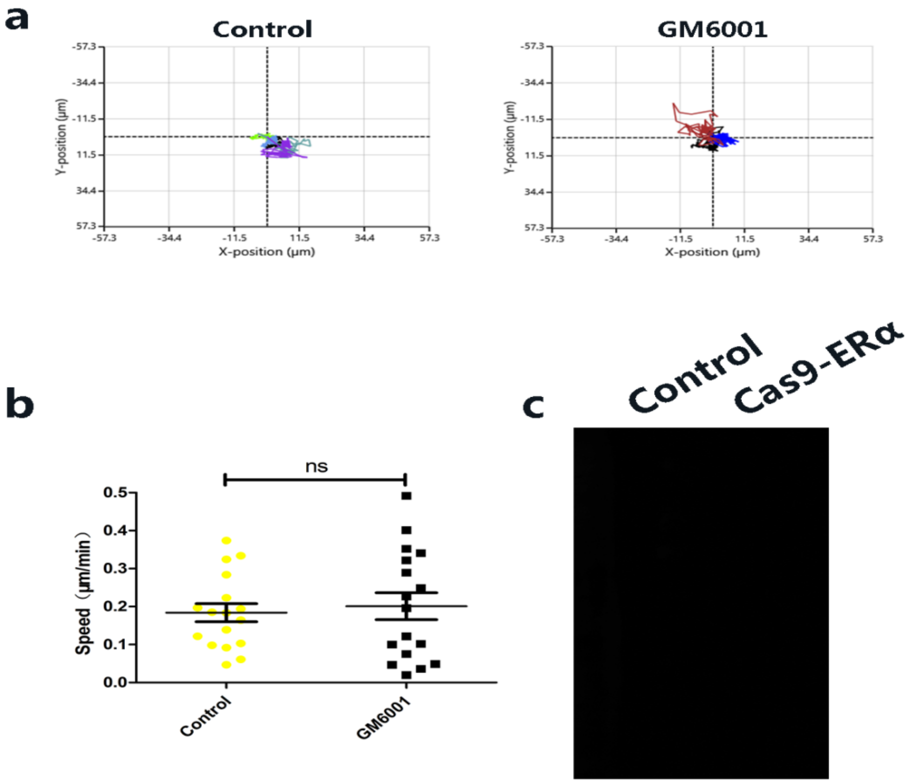

Supplementary Figure 2

Supplementary Figure 2: Loss of ERα induces the amoeboid migration of MCF-7 cells. Related to figure 3. (**a**) Representative tracks of control MCF-7 cells and GM6001 treated MCF-7 cells (**b**) The migration speed of control MCF-7 cells and GM6001 treated MCF-7 cells in the 3D matrix (n=17 cells). (**c**) Conditioned serum-free medium of control or Cas9-ERα MCF-7 cells was collected and used for MMP-2 and MMP-9 activity assessment. (**b**) Graphs show mean ± s.e.m. ns P>0.05 (**b**) Unpaired t-test.

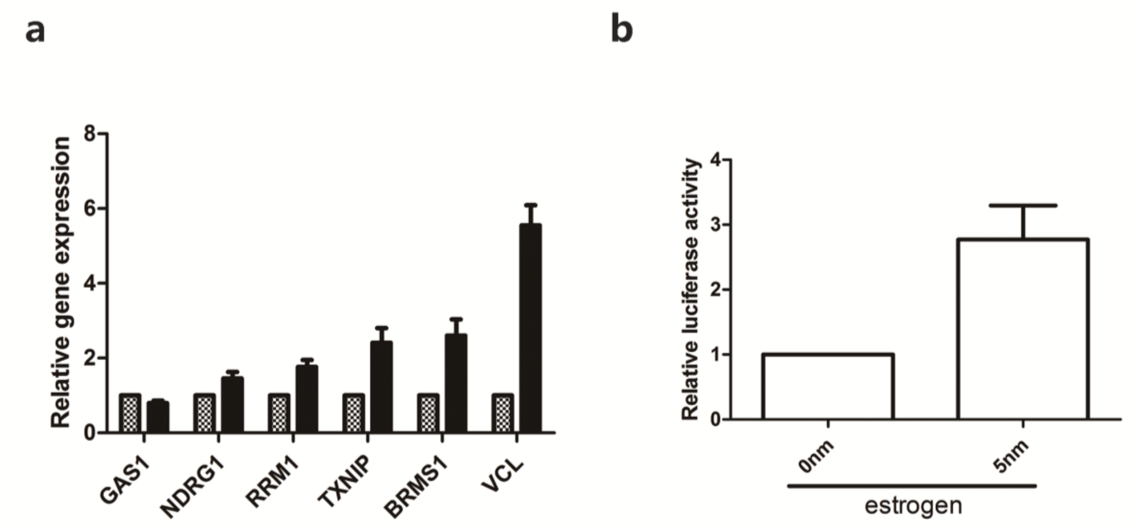

Supplementary Figure 3

Supplementary Figure 3: ERα is a transcriptional promoter of vinculin. Related to figure 4. (a) Real-time PCR was performed to identify gene expression. The main metastasis-associated genes from RNA-seq sequencing were verified by Real-time PCR assay. (**b**) Luciferase activity of vinculin promoter was measured in MCF-7 cells that were treated with 0nm or 5nm estrogen.

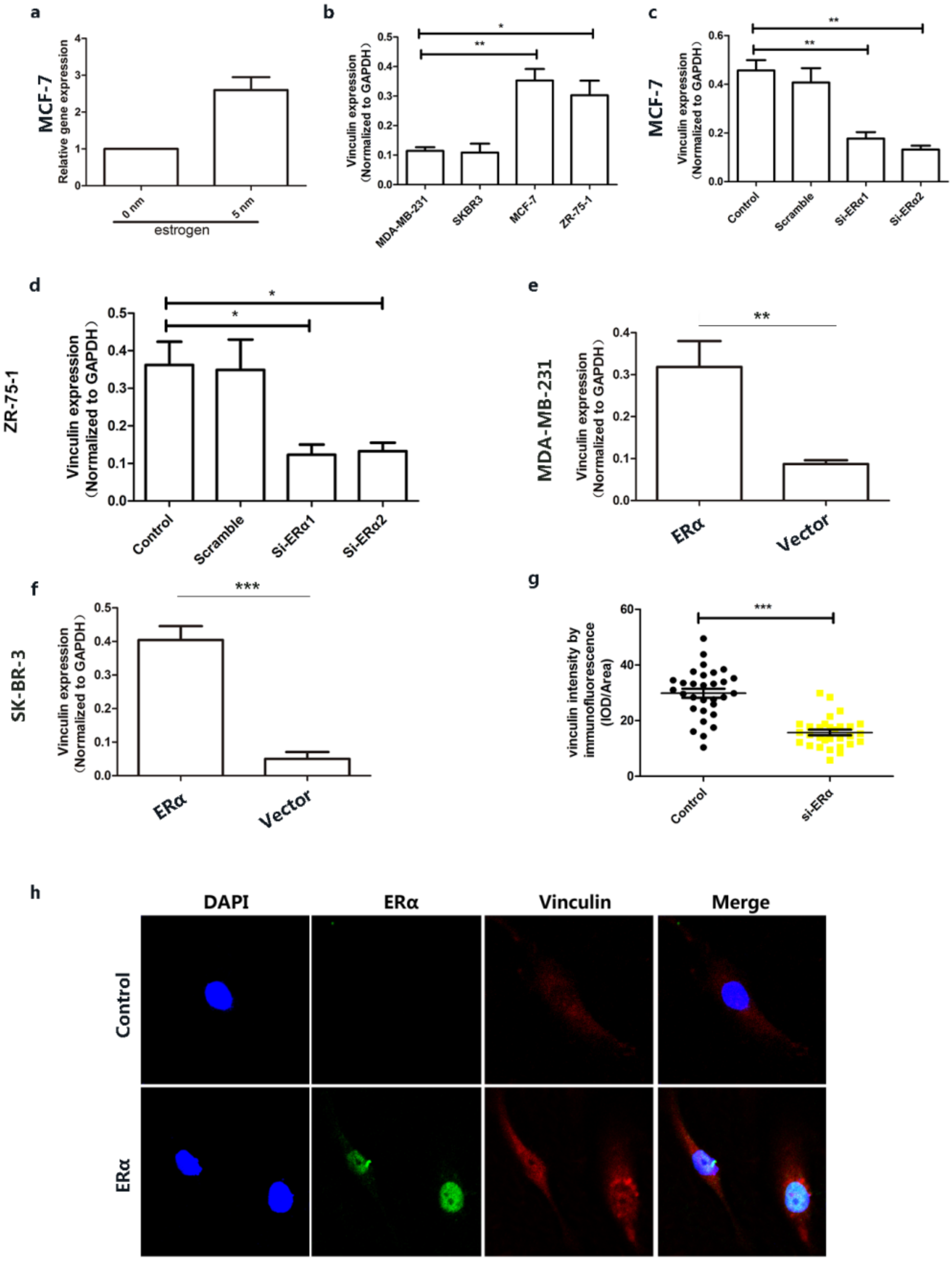

Supplementary Figure 4

Supplementary Figure 4: ERα up-regulates the expression of vinculin in breast cancer cells. Related to figure 5. (a) Real-time PCR detecting the transcription levels of vinculin in MCF-7 cells that were treated with 0nm or 5nm estrogen. The results

were normalized to GAPDH (n=3). (**b**) Quantification of vinculin expression in four breast cancer cell lines normalized to GAPDH (n=3). (**c**) Quantification of vinculin expression in MCF-7 cells normalized to GAPDH (n=3). (**d**) Quantification of vinculin expression in ZR-75-1 cells normalized to GAPDH (n=3). (**e**) Quantification of vinculin expression in MDA-MB-231 cells normalized to GAPDH (n=3). (**f**) Quantification of vinculin expression in SK-BR-3 cells normalized to GAPDH (n=3). (**g**) Quantification of vinculin expression levels from confocal images of fig.5f (n = 30 cells). (**h**) Confocal assay for ERα localization and vinculin expression in MDA-MB-231 (control or ERα-overexpressing) cells. (**a**, **b, c, d, e, f, g**) Graphs show mean ± s.e.m. **P*<0.05 ***P*<0.01 ****P*<0.001. (**b, c, d)** ANOVA with Dunnett *t* test; (**e, f, g**) Unpaired *t*-test.

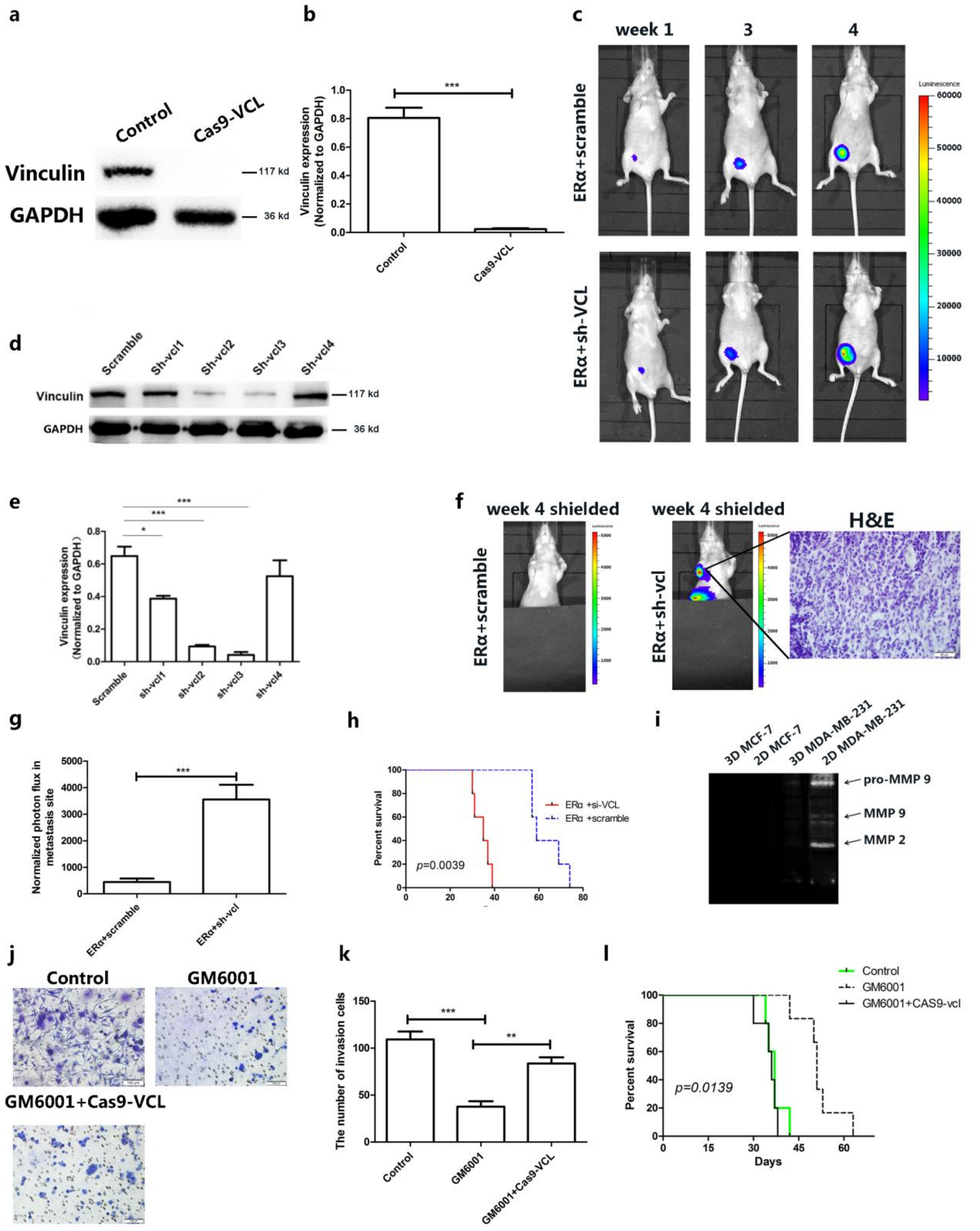

Supplementary Figure 5

Supplementary Figure 5: Vinculin, downstream of ERα, is important for breast cancer metastasis. Related to figure 6. (**a**) Western blot assay for detecting the expression of vinculin in control or Cas9-VCL MCF-7-Luc2 cells. (**b**) Quantification of vinculin expression normalized to GAPDH (n=3). ERα-overexpressing

MDA-MB-231-luc2 cells infected with lentivirus containing vinculin sh-RNA (sh-vcl) or scrambled RNA (scramble) were injected into nude mice to generate xenografts (n=5). (**c**) Bioluminescence imaging at different time points was used to evaluate tumor progression. (**d**) Western blot assay for testing the interference efficiency of vinculin shRNAs. (**e**) Quantification of vinculin expression normalized to GAPDH (n=3). (**f**) Representative images of the scramble group or the sh-vcl group at week 4 are shown after shielding the primary tumor. The lymphatic metastases were determined by H&E staining. (**g**) Luciferase counts in the metastasis sites of (c) at week 4. (**h**) The lifetime of mice injected with scramble or sh-vcl cells. (**i**) Conditioned serum-free 2D or 3D medium of MDA-MB-231 cells was collected and used for MMP-2 and MMP-9 activity assessment; that of MCF-7 cells was used as a negative control. (**j**) A transwell assay was performed in MDA-MB-231 cells, GM6001-treated MDA-MB-231 cells or GM6001-treated and CRISPR/Cas9-mediated *VCL* deleted MDA-MB-231 cells. (**k**) Quantification of invasive cells from (j) (n=3). (**l**) The lifetime of mice injected with control, GM6001-treated control or Cas9-vinculin MDA-MB-231-luc2 cells. (**b, e, g, k**) Graphs show mean ± s.e.m. *P<0.05 **P<0.01 ***P<0.001. (**b, g**) Unpaired t-test; (**e**) ANOVA with Dunnett t test; (**k**) ANOVA with Tukey's post hoc test; (**h, l**) Log-rank test.

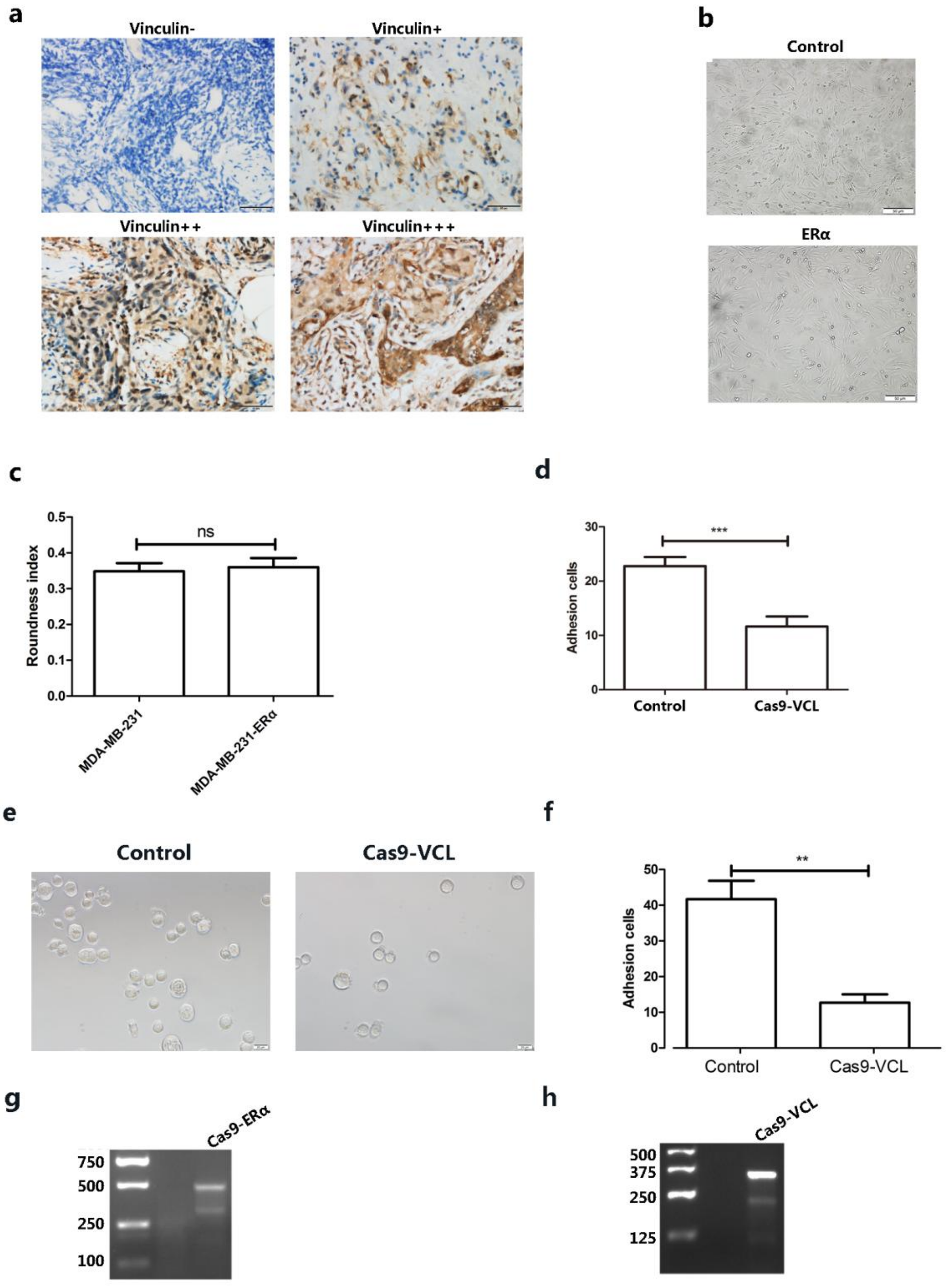

Supplementary Figure 6

Supplementary Figure 6: (a) Immunohistochemistry was performed using a specific antibody against vinculin. Representative images of vinculin expression levels were shown. Scale bars represent 50 μm (40×) (**b**) Optical microscope was used to observe

the morphology of MDA-MB-231 cells or MDA-MB-231-ERα in 2D substrate. Scale bar represents 50 μm (40×). (**c**) Cell morphology (roundness index) of control or ERα-expressing MDA-MB-231 cells (n=100 cells). (**d**) Quantification of breast cancer cell adhesion to Matrigel after vinculin deletion (n=3). (**e**) Representative images of control MCF-7 cells and Cas9-vinculin MCF-7 cells adhering to E-cadherin-coated substrates from optical microscope. Scale bars represent 20 μm (**f**) Quantification of MCF-7 cells adhering to E-cadherin-coated substrates (n=3). (**g**) Validation of the sgRNA directed against the exon of *ESR1* gene using the Knockout and Mutation Detection Kit. (**h**) Validation of the sgRNA directed against the exon of *VCL* gene using the Knockout and Mutation Detection Kit. (**c, d, f**) Graphs show mean ± s.e.m. ns $P>0.05$, ** $P<0.01$, ***$P<0.001$; (**c, d, f**) Unpaired *t*-test.

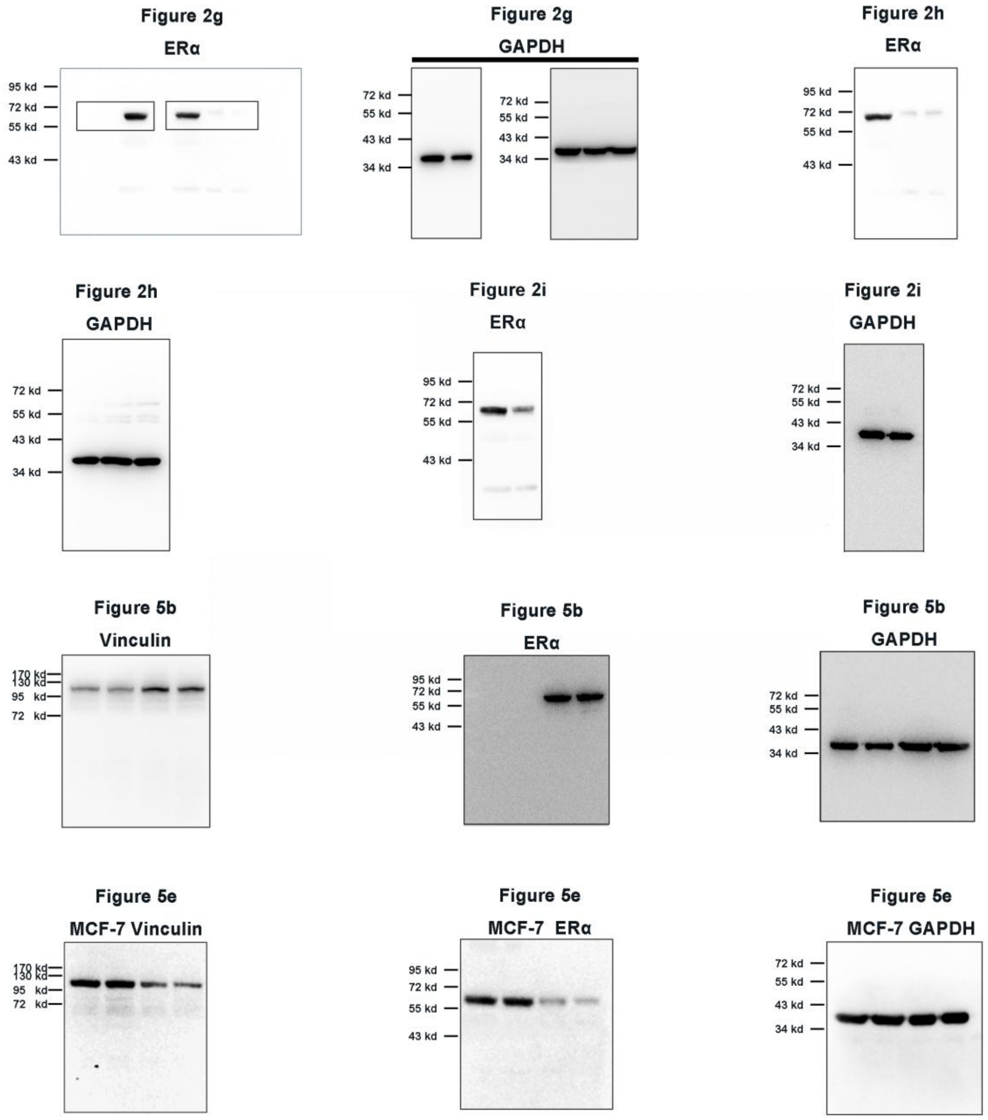

Supplementary Figure 7

Supplementary Figure 7: Original full scans of Western blots related to respective figures as indicated.

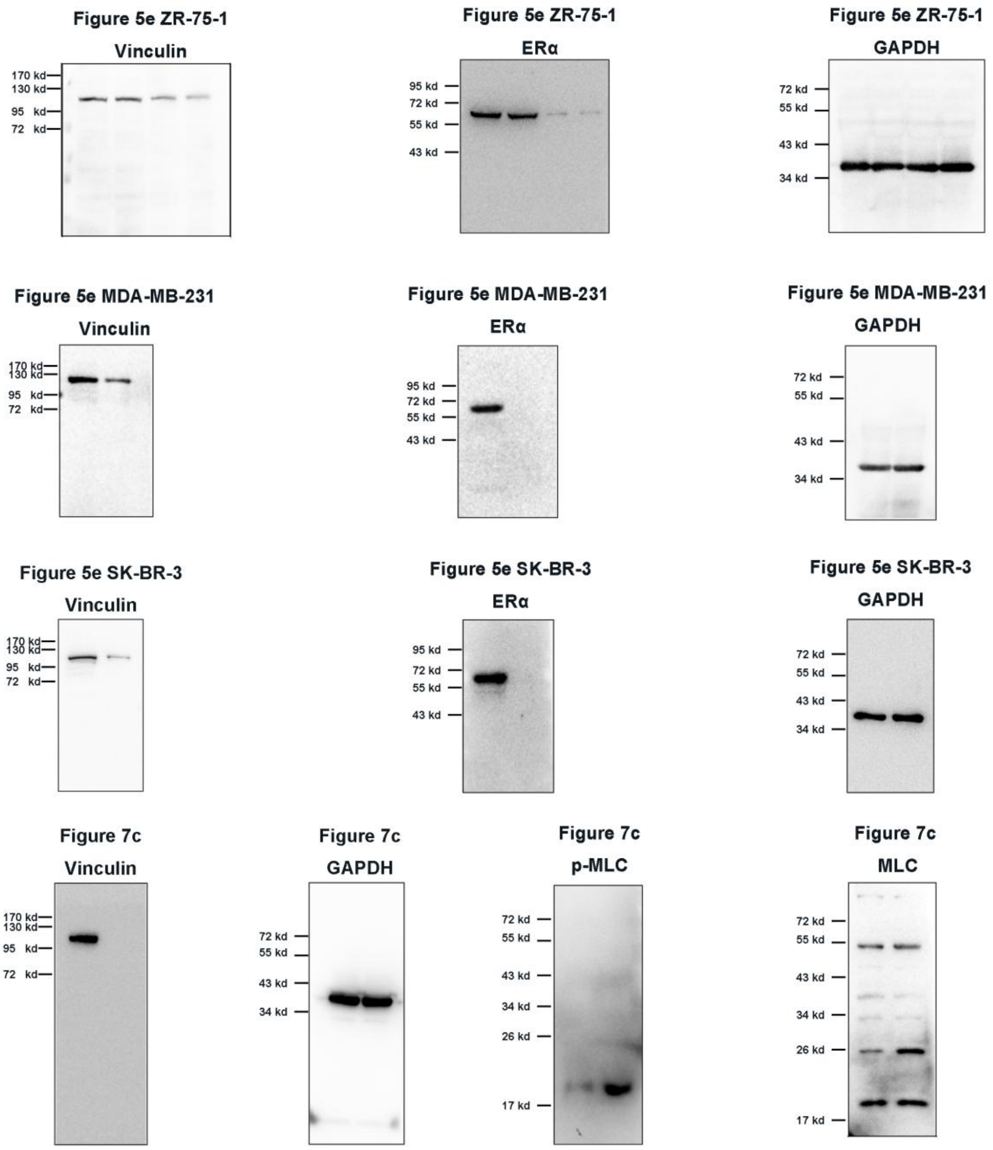

Supplementary Figure 8

Supplementary Figure 8: Original full scans of Western blots related to respective figures as indicated.

Supplementary Tables:

Supplementary Table 1 The expression level of ERα in primary tumor and lymph node metastasis.

ERα Expression level	-	+	++	+++
Primary tumor	0	35	49	40
Lymphatic metastasis	68	21	18	17

Supplementary Table 2 Association between loss of ERα expression and breast carcinoma characteristics

**Variables**	**Total**	**Loss of ERα in lymphatic metastasis**		***P*-value**
		+	-	
	**N=124**	**N=68**	**N=56**	
Age(year)				
＞50	68	32	36	0.055
≤ 50	56	36	20	
Total	124	68	56	
Tumor size				
＞2 cm	68	36	32	0.640
≤ 2 cm	56	32	24	
Total	124	68	56	
Clinical stages				
AJCC Ⅰ	20	6	14	**0.007**
AJCC Ⅱ	53	28	25	
AJCC Ⅲ	51	34	17	
Total	124	68	56	
No.of node metastasis				
N1: 1 to 3	73	33	40	**0.011**
N2: 4 to 9	37	25	12	
N3: ≥10	14	10	4	
Total	124	68	56	
PR status				
PR+	96	48	48	**0.045**
PR-	28	20	8	
Total	124	68	56	
HER2 status				
HER2+	92	52	40	0.523
HER2-	32	16	16	
Total	124	68	56	

Statistical analysis of “Age, Tumor size, PR status, HER2 status” was performed with the chi-square test; Statistical analysis of “Clinical stages, No.of node metastasis” was performed with the Wilcoxon rank sum test.

Supplementary Table 3 The main significantly altered metastasis-associated genes in RNA-seq

Symbol	GeneID	Locus	FPKM (T1+T2/2)	FPKM ((C1+C2/2)	fold_change（T／S）	p_value	fdr	Full Name	Biological _Process	Cellular _Component	Molecular _Function
GAS1	2619	chr9:86944362-86947189	3.4752	1.3227	2.627353141	0.0093653	0.999958	growth arrest-specific 1	GO0000019: negative regulation of protein processing || GO0000019: cellular response to vascular endothelial growth factor stimulus ||GO0000019: regulation of ER to Golgi vesicle-mediated transport	GO:0000033: Plasmid membrane	GO0000033: negative regulation of mitotic cell cycle||GO0000037: cell cycle arrest
NDRG1	10397	chr8:134249413-134309547	13.0272	4.9679	2.622275006	0.0080597	0.999958	N-myc downstream regulated 1	GO:0010038:response to metal ion || GO:0030330:DNA damage response, signal transduction by p53 class mediator || GO:0032287:peripheral nervous system myelin maintenance || GO:0045576:mast cell activation || GO:0071456:cellular response to hypoxia || GO:0090232:positive regulation of spindle	GO:0005634:nucleus || GO:0005737:cytoplasm || GO:0005813:centrosome || GO:0005829:cytosol || GO:0005874:microtubule || GO:0005886:plasma membrane || GO:0005913:cell-cell adherens junction	GO:0005515: protein binding || GO:0008017: microtubule binding || GO:0017137: Rab GTPase binding || GO:0043015: gamma-tubulin binding || GO:0045296: cadherin

									checkpoint	|| GO:0015630:microtubule cytoskeleton || GO:0048471:perinuclear region of cytoplasm || GO:0055038:recycling endosome membrane	binding
RRM1	6240	chr11:4115923-4160106	59.7513	18.3331	3.259203299	0.0238978	0.999958	ribonucleotide reductase M1	GO:0006260:DNA replication || GO:0006260:DNA replication || GO:0009263:deoxyribonucleotide biosynthetic process || GO:0015949:nucleobase-containing small molecule interconversion || GO:0044281:small molecule metabolic process || GO:0051290:protein heterotetramerization || GO:0055086:nucleobase-containing small molecule metabolic process	GO:0005654:nucleoplasm || GO:0005829:cytosol || GO:0005971:ribonucleoside-diphosphate reductase complex	GO:0004748:ribonucleoside-diphosphate reductase activity, thioredoxin disulfide as acceptor || GO:0004748:ribonucleoside-diphosphate reductase activity, thioredoxin disulfide as acceptor || GO:0005515:

											protein binding || GO:0005524: ATP binding
TXNIP	10628	chr1:145438461-145442628	119.403	38.3675	3.112087053	0.0116484	0.999958	thioredoxin interacting protein	GO:0000122:negative regulation of transcription from RNA polymerase II promoter || GO:0006351:transcription, DNA-dependent || GO:0006606:protein import into nucleus || GO:0007049:cell cycle || GO:0009612:response to mechanical stimulus || GO:0009749:response to glucose stimulus || GO:0030216:keratinocyte differentiation || GO:0032355:response to estradiol stimulus || GO:0032570:response to progesterone stimulus || GO:0035872:nucleotide-binding domain, leucine rich repeat containing receptor signaling pathway || GO:0042127:regulation of cell	GO:0005634:nucleus || GO:0005737:cytoplasm || GO:0005758:mitochondrial intermembrane space || GO:0005829:cytosol	GO:0004857: enzyme inhibitor activity || GO:0005515: protein binding || GO:0031625: ubiquitin protein ligase binding

									proliferation || GO:0042493:response to drug || GO:0042542:response to hydrogen peroxide || GO:0043065:positive regulation of apoptotic process || GO:0045087:innate immune response || GO:0048008:platelet-derived growth factor receptor signaling pathway || GO:0051592:response to calcium ion || GO:0051782:negative regulation of cell division || GO:0071228:cellular response to tumor cell		
BRMS1	25855	chr11:66104803-66112582	47.0243	3.9689	11.84819471	0.0080737	0.999958	breast cancer metastasis suppressor 1	GO:0006351:transcription, DNA-dependent || GO:0006915:apoptotic process || GO:0032088:negative regulation of NF-kappaB transcription factor activity || GO:0045892:negative regulation of transcription, DNA-dependent ||	GO:0005634:nucleus || GO:0005737:cytoplasm	GO:0005515: protein binding || GO:0051059: NF-kappaB binding

									GO:0090312:positive regulation of protein deacetylation || GO:2000210:positive regulation of anoikis		
VCL	7414	chr10:75757871-75879914	35.7643	12.5256	2.855296353	0.00804989	0.999952	vinculin	GO:0002009:morphogenesis of an epithelium || GO:0002576:platelet degranulation || GO:0006928:cellular component movement || GO:0006936:muscle contraction || GO:0007155:cell adhesion || GO:0007160:cell-matrix adhesion || GO:0007596:blood coagulation || GO:0030032:lamellipodium assembly || GO:0030168:platelet activation || GO:0030336:negative regulation of cell migration || GO:0034333:adherens junction assembly || GO:0034394:protein localization to cell surface ||	GO:0001725:stress fiber || GO:0005576:extracellular region || GO:0005829:cytosol || GO:0005856:cytoskeleton || GO:0005884:actin filament || GO:0005886:plasma membrane || GO:0005911:cell-cell junction || GO:0005912:adherens junction || GO:0005913:cell-cell adherens junction || GO:0005916:fascia adherens ||	GO:0002162: dystroglycan binding || GO:0003779: actin binding || GO:0005198:structural molecule activity || GO:0005515: protein binding || GO:0008013: beta-catenin binding || GO:0017048: Rho GTPase binding || GO:0045294: alpha-catenin binding ||

									GO:0043297:apical junction assembly || GO:0090136:epithelial cell-cell adhesion	GO:0005925:focal adhesion || GO:0005925:focal adhesion || GO:0030055:cell-substrate junction || GO:0043034:costamere || GO:0043034:costamere || GO:0043234:protein complex	GO:0045296: cadherin binding

Supplementary Table 4 The expression level of ERα was positively correlated with the vinculin expression level in the primary tumor

		Vinculin				
	Primary tumor	-	+	++	+++	Total
ERα	+	5	19	8	3	35
	++	1	17	26	5	49
	+++	1	5	9	25	40
	Total	7	41	43	33	124

Spearman rank correlation analysis was used. (P<0.001, R^2=0.528)

Supplementary Table 5 The expression level of ERα was positively correlated with the vinculin expression level in the lymph node metastasis

		Vinculin				
	lymphatic metastasis	-	+	++	+++	Total
ERα	-	17	28	13	10	68
	+	3	13	4	1	21
	++	0	3	11	4	18
	+++	1	3	3	10	17
	Total	21	47	31	25	124

Spearman rank correlation analysis was used. (P<0.001, R^2=0.366)

Supplementary Table 6 The sequence of ERα siRNAs

ID	sense（5'-3'）	antisense（5'-3'）
ESR1-homo-1	5' CAGGCCAAAUUCAGAUAAUTT 3'	5' AUUAUCUGAAUUUGGCCUGTT 3'
ESR1-homo-2	5' GGUCCACCUUCUAGAAUGUTT 3'	5' ACAUUCUAGAAGGUGGACCTT 3'
ESR1-homo-3	5' GAGGGAGAAUGUUGAAACATT 3'	5' UGUUUCAACAUUCUCCCUCTT 3'
Negative control	5' UUCUCCGAACGUGUCACGUTT 3'	5' ACGUGACACGUUCGGAGAATT 3'

Supplementary Table 7 The sequence of vinculin shRNAs

ID	5’	stem	loop	stem	3’
VCL-RNAi(1)-a	Ccgg	gcACAGATAAACGGATTAGAA	CTCGAG	TTCTAATCCGTTTATCTGTGC	TTTTTg
VCL-RNAi(1)-b	aattcaaaaa	gcACAGATAAACGGATTAGAA	CTCGAG	TTCTAATCCGTTTATCTGTGC	
VCL-RNAi(2)-a	Ccgg	ccCTGGAAATCAAGCTGCTTA	CTCGAG	TAAGCAGCTTGATTTCCAGGG	TTTTTg
VCL-RNAi(2)-b	aattcaaaaa	ccCTGGAAATCAAGCTGCTTA	CTCGAG	TAAGCAGCTTGATTTCCAGGG	
VCL-RNAi(3)-a	Ccgg	cgGTTGGTACTGCTAATAAAT	CTCGAG	ATTTATTAGCAGTACCAACCG	TTTTTg
VCL-RNAi(3)-b	aattcaaaaa	cgGTTGGTACTGCTAATAAAT	CTCGAG	ATTTATTAGCAGTACCAACCG	
VCL-RNAi(4)-a	Ccgg	gcTCGAGATTATCTAATTGAT	CTCGAG	ATCAATTAGATAATCTCGAGC	TTTTTg
VCL-RNAi(4)-b	aattcaaaaa	gcTCGAGATTATCTAATTGAT	CTCGAG	ATCAATTAGATAATCTCGAGC	

Supplementary Table 8 The sequence of relative sgRNA

sgRNA	sequence
ESR1	CCATCCCAGATGCTTTGGTG
VCL	CCTGCTCCTTACCTTCGATG

Supplementary Table 9 The sequence of primer sets flanking related putative ERα binding sites in the promoter region of vinculin

ID	sense（5'-3'）	antisense（5'-3'）
Primer 1	5' GTT CAC GCC ATT GTC CTG 3'	5' TTA TCC AGT TCC CTC ACG 3'
Primer 2	5' CAA CCC AAG TCC ATG AGT 3'	5' CTG GGT GTG TAG CCT TCT 3'
Primer 3	5' GTT TAC GTG AAT GGG ACG 3'	5' GTT TGA TGA GAC CAA GGG 3'
Primer 4	5' GAG CAT CTC GAA AAG GGA 3'	5' AGA GAC AGA CTG TGC AGC 3'

Supplementary Table 10 The sequence of primer sets for real-time PCR assay

ID	Forward（5'-3'）	Reverse（5'-3'）
ESR1	5' TCTTGGACAGGAACCAGGGA 3'	5' CAGAGACTTCAGGGTGCTGG 3'
Vinculin	5' CACCGTGAAAGAGTTGCTGC 3'	5' TGGCTTCAGTGTCCTTGCTG 3'
GAPDH	5' GTCAAGGCTGAGAACGGGAA 3'	5' AAATGAGCCCCAGCCTTCTC 3'
